# Co-occurrence *CDK4/6* amplification serves as biomarkers of de novo EGFR TKI resistance in sensitizing *EGFR* mutation non-small cell lung cancer

**DOI:** 10.1038/s41598-022-06239-y

**Published:** 2022-02-09

**Authors:** Piyada Sitthideatphaiboon, Chinachote Teerapakpinyo, Krittiya Korphaisarn, Nophol Leelayuwatanakul, Nopporn Pornpatrananrak, Naravat Poungvarin, Poonchavist Chantranuwat, Shanop Shuangshoti, Chatchawit Aporntewan, Wariya Chintanapakdee, Virote Sriuranpong, Chanida Vinayanuwattikun

**Affiliations:** 1 Division of Medical Oncology, Department of Medicine, Faculty of Medicine, Chulalongkorn University and The King Chulalongkorn Memorial Hospital, Bangkok, 10330 Thailand; 2 Chula GenePRO Center, Research Affairs, Chulalongkorn University and The King Chulalongkorn Memorial Hospital, Bangkok, 10330 Thailand; 3 Division of Medical Oncology, Department of Medicine, Faculty of Medicine Siriraj Hospital, Mahidol University, Siriraj, Bangkok Noi, Bangkok, 10700 Thailand; 4 Division of Pulmonary and Critical Care Medicine, Department of Medicine, Faculty of Medicine, Chulalongkorn University and The King Chulalongkorn Memorial Hospital, Bangkok, Thailand; 5 Department of Surgery, Faculty of Medicine, Chulalongkorn University and The King Chulalongkorn Memorial Hospital, Bangkok, 10330 Thailand; 6 Department of Clinical Pathology, Faculty of Medicine Siriraj Hospital, Mahidol University, Siriraj, Bangkok Noi, Bangkok, 10700 Thailand; 7 Department of Pathology, Faculty of Medicine, Chulalongkorn University and The King Chulalongkorn Memorial Hospital, Bangkok, 10330 Thailand; 8 Department of Mathematics and Computer Science & Omics Science and Bioinformatics Center, Faculty of Science, Chulalongkorn University, Bangkok, 10330 Thailand; 9 Department of Radiology, Faculty of Medicine, Chulalongkorn University and The King Chulalongkorn Memorial Hospital, Bangkok, Thailand

**Keywords:** Cancer, Biomarkers, Molecular medicine, Oncology

## Abstract

Despite the development of predictive biomarkers to shape treatment paradigms and outcomes, de novo EGFR TKI resistance advanced non-small cell lung cancer (NSCLC) remains an issue of concern. We explored clinical factors in 332 advanced NSCLC who received EGFR TKI and molecular characteristics through 65 whole exome sequencing of various EGFR TKI responses including; de novo (progression within 3 months), intermediate response (IRs) and long-term response (LTRs) (durability > 2 years). Uncommon *EGFR* mutation subtypes were significantly variable enriched in de novo resistance. The remaining sensitizing *EGFR* mutation subtypes (exon 19 del and L858R) accounted for 75% of de novo resistance. Genomic landscape analysis was conducted, focusing in 10 frequent oncogenic signaling pathways with functional contributions; cell cycle, Hippo, Myc, Notch, Nrf2, PI-3-Kinase/Akt, RTK-RAS, TGF-β, p53 and β-catenin/Wnt signaling. Cell cycle pathway was the only significant alteration pathway among groups with the FDR *p*-value of 6 × 10^–4^. We found only significant *q*-values of < 0.05 in 7 gene alterations; *CDK6*, *CCNE1, CDK4, CCND3, MET, FGFR4* and *HRAS* which enrich in de novo resistance [range 36–73%] compared to IRs/LTRs [range 4–22%]. Amplification of *CDK4*/6 was significant in de novo resistance, contrary to IRs and LTRs (91%, 27.9% and 0%, respectively). The presence of co-occurrence *CDK4/6* amplification correlated with poor disease outcome with HR of progression-free survival of 3.63 [95% CI 1.80–7.31, *p*-value < 0.001]. The presence of *CDK4/6* amplification in pretreatment specimen serves as a predictive biomarker for de novo resistance in sensitizing *EGFR* mutation.

## Introduction

The discovery of molecularly targeted therapies has dramatically changed the paradigm of treatment in advanced non-small cell lung cancer (NSCLC). Activating epidermal growth factor receptor (*EGFR*) mutations are key drivers of NSCLC and are more common in East Asian than non-Asian populations (40–60% vs. 10–15%, respectively)^1–3^. Several randomized studies support the use of EGFR tyrosine kinase inhibitors (TKIs) as the standard first-line treatment for patients with activating *EGFR* mutations^4–11^. However, there is heterogeneity in treatment responses of EGFR TKIs with a progression-free survival (PFS) ranging from a few months to several years and resistance disease inevitably emerges. Approximately 10–20% of patients that harbor activating *EGFR* mutations do not exhibit objective responses to EGFR TKIs. Previous studies have identified molecular mechanisms that are associated with diverse responses to TKIs, including *EGFR* mutation subtype^12,13^, primary existence of the *EGFR* T790M mutation or coexistence of *EGFR* mutations with other genetic alterations^14,15^, for example, *KRAS* mutation, *MET* amplification, *PIK3CA* mutation, inactivation of *TP53*, or *BIM* polymorphism. Taken together, comparing clinical characteristics and comprehensive genomic landscapes via whole exome sequencing (WES) in diverse EGFR TKI responders; de novo resistance, intermediate and long-term responses, *EGFR* mutation NSCLC will enable the identification of potential mechanisms that confer resistance to EGFR TKI treatment.

## Results

### Patient demographics

**Figure 1:**
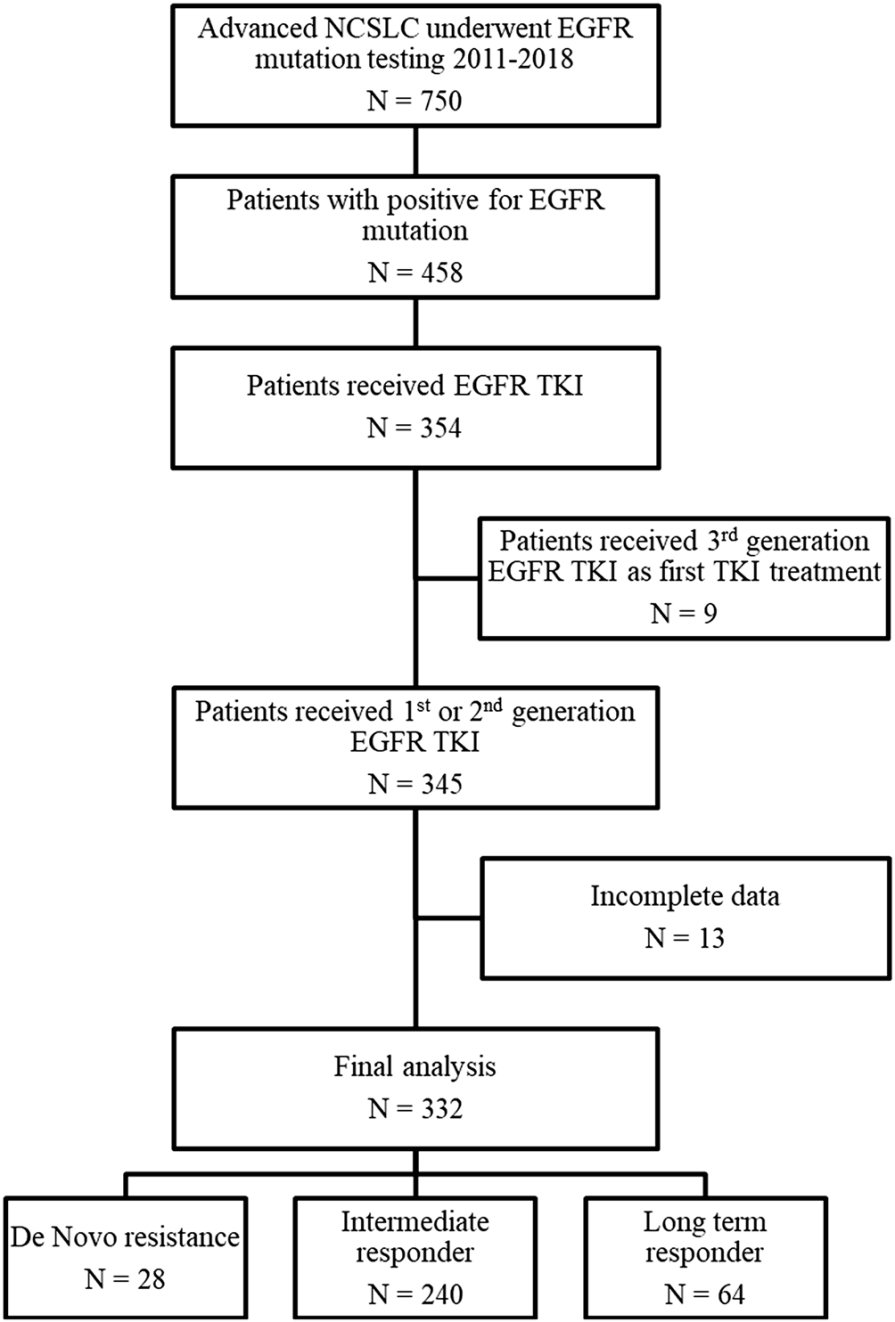
Consort diagram of 332 EGFR mutation-positive recurrence or advanced NSCLC patients in this study.

Of the 458 patients with NSCLC whose tumors harbored *EGFR*-activating mutation, 332 patients received EGFR TKIs and complete follow-up data were included in the final analysis (Fig. 1). Patient demographics are summarized in Table 1. The median age was 64 years (interquartile range [IQR] 54.3 to 72 years). Sixty-four percent of patients were women. Most of the patients were never smokers (80%) and had 0–1 score of ECOG performance status (87%). A majority of the patients were adenocarcinoma (95%), metastatic disease at presentation (76%) and 1–2 metastatic sites (75%). Baseline brain metastases were present in 22% of the overall population. Regarding *EGFR* mutation subtype, 169 patients (51%) harbored exon 19 deletion, 136 patients (41%) harbored L858R, and 27 patients (8%) had other mutations including G719X in exon 18 (N = 6), exon 20 insertion (N = 2), S768I in exon 20 (N = 1), and L861G or Q in exon 21 (N = 6). Twelve patients had any or two coexisting *EGFR* mutations (complex mutation) including one patient with a L858R with a coexisting de novo *EGFR* T790M mutation, respectively. A total of 218 patients (66%) received EGFR TKIs as first-line treatment, and the remaining were treated as the subsequent line treatment. The majority of 1st generation EGFR TKIs were administrated up to 95%, composed of gefitinib 59% and erlotinib 36%. The objective response rate (ORR) of EGFR TKIs in our cohort was 60.5%. The median duration of EGFR TKIs treatment was 12.4 months (IQR 7.1 to 20.5 months), the median duration of response was 10.0 months (IQR, 5.6–16.3 months), the median duration of stable disease (SD) was 9.1 months (IQR, 4.7–16.3 months), and the median PFS for any lines of EGFR-TKI was 12.2 months (95% confidence interval [CI] 11.0–13.3 months). At data cut-off on June 30, 2020, the median follow-up duration was 51.5 months (95% CI 45.9–57.0). A total of 217 patients (65%) ceased. The median OS of the overall study cohort was 32.9 months (95% CI 27.9–37.8 months).

**Table 1:** Patient demographics in the overall population, 332 advanced or recurrent NSCLC who received 1st or 2nd generation EGFR TKI.

Characteristics	All (N = 332)	De novo resistance (N = 28)	Intermediate responders (N = 240)	Long-term responders (N = 64)	*P* values
**Age at diagnosis, n (%)**					0.44
Median (IQR)	64 (54.3–72)	60.5 (50–68)	64 (55–72)	63 (55–72)	
< 60 years	136 (41%)	14 (50%)	99 (41.3%)	23 (35.9%)	
≥ 60 years	196 (59%)	14 (50%)	141 (58.7%)	41 (64.1%)	
**Gender, n (%)**					0.56
Male	118 (35.5%)	12 (42.9%)	86 (35.8%)	20 (31.3%)	
Female	214 (64.5%)	16 (57.1%)	154 (64.2%)	44 (68.8%)	
**ECOG PS, n (%)**					0.07
0–1	272 (86.6%)	21 (75%)	197 (86.4%)	54 (93.1%)	
≥ 2	42 (13.4%)	7 (25%)	31 (13.6%)	4 (6.9%)	
Missing	18		12	6	
**Smoking status, n (%)**					0.10
Never	229 (80.4%)	16 (64%)	168 (82%)	45 (81.8%)	
Current/former	56 (19.6%)	9 (36%)	37 (18%)	10 (18.2%)	
Missing	47	3	35	9	
**Histology, n (%)**					0.48
Adenocarcinoma	310 (95.4%)	28 (100%)	225 (95%)	57 (95%)	
Non adenocarcinoma	15 (4.6%)	0	12 (5%)	3 (5%)	
Missing	7		3	4	
**Stage at diagnosis, n (%)**					0.003*
Recurrent	79 (23.8%)	1 (3.6%)	55 (22.9%)	23 (35.9%)	
Metastatic	253 (76.2%)	27 (96.4%)	185 (77.1%)	41 (64.1%)	
Curative surgery, n (%)	70 (21.1%)	1 (3.6%)	48 (20%)	21 (32.8%)	0.01*
**Number of metastatic sites, n (%)**					0.002*
1–2 sites	248 (74.7%)	20 (71.4%)	169 (70.4%)	59 (92.2%)	
≥ 3 sites	84 (25.3%)	8 (28.6%)	71 (29.6%)	5 (7.8%)	
Brain metastasis, n (%)	73 (22%)	9 (32.1%)	54 (22.5%)	10 (15.6%)	0.20
Liver metastasis, n (%)	37 (11.1%)	2 (7.1%)	29 (12.1%)	6 (9.4%)	0.65
***EGFR* mutation subtypes, n (%)**					< 0.001*
Del19	169 (50.9%)	10 (35.7%)	126 (52.5%)	33 (51.6%)	
L858R	136 (41%)	10 (35.7%)	98 (40.8%)	28 (43.8%)	
G719X	6 (1.8%)	3 (10.7%)	3 (1.3%)	0	
L861G or Q	6 (1.8%)	3 (10.7%)	3 (1.3%)	0	
S768I	1 (0.3%)	0	1 (0.4%)	0	
Exon 20 insertion	2 (0.6%)	1 (3.6%)	1 (0.4%)	0	
Any or combined mutations	12 (3.6%)	1 (3.6%)	8 (3.3%)	3 (4.7%)	
***EGFR* mutation subtypes, n (%)**					< 0.001*
Del19	169 (50.9%)	10 (35.7%)	126 (52.5%)	33 (51.6%)	
L858R	136 (41%)	10 (35.7%)	98 (40.8%)	28 (43.8%)	
Uncommon^a^	15 (4.5%)	7 (25%)	8 (3.3%)	0	
Any or combined mutations	12 (3.6%)	1 (3.6%)	8 (3.3%)	3 (4.7%)	
**Line of EGFR TKIs, n (%)**					0.21
First line	218 (65.7%)	17 (60.7%)	153 (63.8%)	48 (75%)	
Later line	114 (34.3%)	11 (39.3%)	87 (36.3%)	16 (25%)	
**Type of EGFR TKIs, n (%)**					0.01*
Gefitinib	197 (59.3%)	17 (60.7%)	145 (60.4%)	35 (54.7%)	
Erlotinib	121 (36.4%)	8 (28.6%)	84 (35%)	29 (45.3%)	
2nd generation^b^	14 (4.3%)	3 (10.7%)	11 (4.6%)	0	
Duration of TKI treatment, median (IQR, months)	12.4 (7.1–20.5)	2.3 (1.6–3)	11.9 (7.6–15.9)	32.4 (27.8–37.3)	< 0.001*
ORR, n (%)	201 (60.5%)	1 (3.6%)	159 (66.2%)	41 (64.1%)	< 0.001*

^a^Uncommon *EGFR* mutations, including G719X in exon 18 (n = 6), exon 20 insertion (n = 2), S768I in exon 20 (n = 1) and L861G or Q in exon 21 (n = 6). ^b^Afatinib and dacomitinib were used in 13 and 1 patients, respectively.

*Del19* exon 19 deletion, *ECOG PS* Eastern Cooperative Oncology Group performance status, *EGFR* epidermal growth factor receptor, *IQR* interquartile range, *ORR* overall response rate.

### Clinical factors associated with the response of EGFR TKIs

Patients were categorized into 3 groups based on EGFR TKI responsiveness (1) de novo resistance, EGFR TKI resistance who were defined as the best response was progressive disease (PD) or SD less than 3 months while receiving EGFR TKIs. This group represented intrinsic resistance to EGFR TKIs^16^; (2) intermediate responder, developed acquired resistance to EGFR TKIs according to the proposed criteria by Jackman^17^ and (3) long-term responder, durable disease control with EGFR TKIs more than 2 years^18^. Patient characteristics of the three groups were listed in Table 1.

The de novo resistance groups were significantly associated with metastatic disease at presentation (96.4%; *p*-value 0.003) and uncommon *EGFR* mutation subtype (25%; *p*-value 0.001). Presence of metastatic disease at diagnosis was found in 77% and 64% of IRs and LTRs, respectively. Only 3.3% of tumors harboring uncommon *EGFR* mutations were found in IRs and absent in LTRs. There was no significant difference in age, gender, PS, smoking status, histology, baseline liver or brain metastasis, and the treatment lines of TKI between the three groups. Moreover, there was no difference in EGFR TKIs response between the exon 19 deletion and L858R mutation.

Logistic regression was performed to evaluate the correlation of clinical variables and response to EGFR TKIs (Table 2, Table S1) which revealed uncommon *EGFR* mutation subtype as significant variables in de novo resistance vs. IRs and LTRs with odds ratios (OR) of 6.83 ([95% CI 2.36–19.80], *p*-value < 0.001) and 16.84 ([95%CI 1.66–171.45, *p*-value 0.02), respectively. Poor ECOG performance status and metastatic disease at presentation were independent factors in de novo resistance vs. LTRs with OR of 7.39 ([95%CI 1.16–47.16, *p*-value = 0.04) and 61.45 ([95% CI 1.29–not estimated], *p*-value = 0.04), respectively (Table 2). These results were consistent with the results of multivariate Cox’s proportional hazards analysis which revealed that those factors were correlated with PFS of EGFR TKIs (Table S2). Kaplan–Meier analysis, according to each clinical variable, correlated with PFS/OS and results were shown in Fig. S1 and Fig. S2. Survival analysis and subsequent treatment were described in the supplementary information (Table S3-S6).

**Table 2:** Univariate and multivariate analyses of clinical variables and response to EGFR TKIs.

De novo resistance versus IRs variables^a^	Univariate		Multivariate	
	OR (95% CI)	P value	OR (95% CI)	P value
Age (< 60/ ≥ 60)	1.42 (0.65–3.12)	0.38		
Sex (male/female)	1.34 (0.61–2.97)	0.47		
ECOG PS (≥ 2/0–1)	2.12 (0.83–5.40)	0.12		
Smoking (current-former/never)	2.55 (1.05–6.23)	0.04*	2.14 (0.83–5.54)	0.12
Histology (non-ADC/ADC)	Not estimated	1		
Stage at diagnosis (M1/M0)	8.03 (1.07–60.42)	0.04*	5.01 (0.65–38.91)	0.12
Curative surgery (no/yes)	3.25 (0.75–14.17)	0.12		
Number of metastatic sites (≥ 3/1–2)	0.95 (0.40–2.26)	0.91		
Brain metastasis (yes/no)	1.63 (0.70–3.81)	0.26		
Liver metastasis (yes/no)	0.56 (0.13–2.48)	0.45		
*EGFR* subtype (others/common)	5.60 (2.14–14.69)	< 0.001*	6.83 (2.36–19.80)	< 0.001*
Common *EGFR* subtype (Del19/L858R)	0.78 (0.31–1.94)	0.59		
Line of TKI (first/later)	0.88 (0.39–1.96)	0.75		
Generation of TKI (first/second)	0.40 (0.11–1.53)	0.18		
First generation of TKI (gefitinib/erlotinib)	1.23 (0.51–2.98)	0.64		

De novo resistance versus LTRs variables^a^	Univariate		Multivariate	
	OR (95% CI)	P value	OR (95% CI)	P value
Age (< 60/ ≥ 60)	1.78 (0.73–4.38)	0.21		
Sex (male/female)	1.65 (0.66–4.13)	0.28		
ECOG PS (≥ 2/0–1)	4.50 (1.19–16.98)	0.03*	7.39 (1.16–47.16)	0.04*
Smoking (current-former/never)	2.53 (0.87–7.35)	0.09	2.38 (0.55–10.33)	0.25
Histology (non-ADC/ADC)	Not estimated	1		
Stage at diagnosis (M1/M0)	15.15 (1.93–118.86)	0.01*	61.45 (1.29–NE)	0.04*
Curative surgery (no/yes)	6.35 (1.38–29.32)	0.02*	0.30 (0.02–5.55)	0.42
Number of metastatic sites (≥ 3/1–2)	4.72 (1.38–16.10)	0.01*	4.05 (0.54–30.53)	0.18
Brain metastasis (yes/no)	2.56 (0.90–7.25)	0.08	1.92 (0.42–8.67)	0.40
Liver metastasis (yes/no)	0.74 (0.14–3.93)	0.73		
*EGFR* subtype (others/common)	8.13 (1.97–33.64)	0.004*	16.84 (1.66–171.45)	0.02*
Common *EGFR* subtype (Del19/L858R)	0.85 (0.31–2.33)	0.75		
Line of TKI (first/later)	0.52 (0.20–1.33)	0.17		
Generation of TKI (first/second)	Not estimated	1		
First generation of TKI (gefitinib/erlotinib)	1.76 (0.67–4.66)	0.26		

^a^Category after the slash (/) was set as reference category.

*ADC* adenocarcinoma, *Del19* exon 19 deletion, *ECOG PS* Eastern Cooperative Oncology Group performance status, *EGFR* epidermal growth factor receptor, *IRs* intermediate responders, *LTRs* long-term responders, *M0* recurrent disease, *M1* metastatic disease, *TKI* tyrosine kinase inhibitor.

### Comparative “cohort-normal” vs. “match-normal” WES analysis workflow in exploratory cohort 65 tumor-normal resectable lung cancer

To define the concordance variant calling between “cohort-normal” and “match-normal”, we conducted WES analysis in 65 patients with resectable stage adenocarcinoma of the lung who underwent surgery as a curative intent. A “cohort-normal” pipeline was conducted using in-house normal reference obtained from either leucocyte or normal lung. In general, mutation profiling for 21 driver genes and CNAs (arm-level and focal-level) were consistent with the lung adenocarcinoma East Asian cohort^19^ (Fig. S5). The most frequent driver mutations were *EGFR* (60%), *TP53* (28%) and *RMB10* (11%), consistent with the East Asian cohort (47%, 36% and 8%, respectively), while the *KRAS* mutation was found 4% lower than the East Asian cohort (11%). Median TMB (including synonymous and non-synonymous mutations) was low at 1.84 Mb^−1^ (range: 0.24–25.14 Mb^−1^) which is a dominant characteristic of the majority of never smoker, adenocarcinoma lung cancer (73%) in our study. Many focal CNAs were found around driver gene amplification in *EGFR*, *MYC*, *MDM2*, *KRAS* and *CCNE1* as well as deletion in *ARID1A* and *APC* (Fig. S6A and S6B). Somatic prediction in “cohort-normal” workflow was conducted using PureCN based on altered allelic fractions of germline and somatic variants which previously showed median accuracy of somatic variants of 97.2% in TCGA-LUAD^20^. There was a high correlation (*R* = 0.99, *p*-value < 2.2 × 10^–16^) of all non-synonymous mutations between “cohort-normal” and “match-normal” workflow (Fig. S7B). There were 3,445 non-synonymous variants in the “cohort-normal” workflow and 4,717 non-synonymous variants in the “match-normal” workflow. Eighty-four percent of all non-synonymous mutations in “cohort-normal” were concordant to 61.3% of “match-normal” workflow. The concordant rates in “cohort-normal” were 89% and 92.3% in 307 significant genes from LUAD 7 studies (additional information: Table S12) and 206 genes from 10 significant pathway analysis, respectively^21^ (Fig. S7A). Cohort-normal workflow of the non-synonymous mutation variant with high-concordance rate to match-normal workflow in 206 genes was adopted in WES analysis of 65 *EGFR* mutation-positive recurrence or advanced NSCLC. The retained variants according to the filtered algorithm is shown in Fig. S8. Demographic characteristics of 65 resectable stage adenocarcinoma of lung were shown in additional information: Table S11.

### Elucidation of molecular analysis correlation with the response of EGFR TKI in “cohort-normal”

We selected participants for genomic study based on retrospective aforementioned-response classification. Demographic characteristics, response treatment of 65 advanced stage NSCLC, received EGFR TKIs who had adequate tissue for WES were shown in Table 3, Fig S3. We analyzed exome sequencing with target sequences of approximately 90 Mb. The average depth of coverage within targets was 65× (range 60–94×) with 95% of targeted bases were covered by at least 10 reads. Based on the “cohort-normal” algorithm, 14,508 non-synonymous variants were retained from 65 WES recurrence or advanced *EGFR* mutation-positive NSCLC (additional information: Table S7). Median non-synonymous mutation was 2.3 Mb^−1^ (range 1.5–6.0 Mb^−1^). The median frequency of non-synonymous mutation in de novo resistance was 1.15 Mb^−1^ (range 0.65–3.33 Mb^−1^) lower than IRs and LTRs which was 2.82 Mb^−1^ (range 1.07–6.02 Mb^−1^, *p*-value < 0.001) and 1.77 Mb^−1^ (range 1.18–2.98, *p*-value 0.01). However, this might be an effect of lower average read depth in de novo resistance than IRs and LTRs, 53× (range 40–63×), 68× (range 60–94×, *p*-value < 0.001) and 64.3× (range 40–80×, *p*-value 0.03) (Fig. S9A). No statistical difference was present in tumor ploidy nor tumor purity among 3 groups. Median tumor ploidies were 3.6 (range 1.9–4.6) in de novo resistance, 2.4 (range 1.0–4.6, *p*-value 0.058) in IRs and 2.6 (range 1.6–3.8, *p*-value 0.055) in LTRs (Fig. S9B). Median tumor purities were 0.38 (range 0.23–0.59) in de novo resistance, 0.33 (range 0.18–0.66, *p*-value = 0.33) in IRs and 0.37 (range 0.26–0.61, *p*-value = 0.94) in LTRs (Fig. S9C). Significant CNAs of advanced 65 WES recurrence or advanced NSCLC and gene-level segment integer copies number estimation are shown in additional information: Table S8.

**Table 3:** Demographic characteristic of 65 advanced or recurrence NSCLC participants who performed WES.

Characteristics	All (N = 65)	De novo resistance (N = 11)	Intermediate responders (N = 44)	Long-term responders (N = 10)
**Age at diagnosis, n (%)**				
< 60 years	28 (43%)	6 (54.6%)	18 (40.9%)	4 (40%)
≥ 60 years	37 (57%)	5 (45.4%)	26 (59.1%)	6 (60%)
**Gender, n (%)**				
Male	20 (30.7%)	3 (27.2%)	15 (34%)	2 (20%)
Female	45 (69.3%)	8 (72.8%)	29 (66%)	8 (80%)
**Smoking status, n (%)**				
Never	54 (83%)	9 (81.9%)	35 (79.5%)	10 (100%)
Current/former	11 (17%)	2 (18.1%)	9 (20.5%)	0
**Histology, n (%)**				
Adenocarcinoma	61 (93.8%)	11 (100%)	40 (90.9%)	10 (100%)
NSCLC NOS	4 (6.2%)	0	4 (9.1%)	0
**Stage at diagnosis, n (%)**				
Recurrent	10 (15.3%)	1 (9.1%)	8 (18.2%)	1 (10%)
Metastatic	55 (84.7%)	10 (90.9%)	36 (81.8%)	9 (90%)
***EGFR* mutation subtypes, n (%)**				
Del19	36 (55.3%)	7 (63.6%)	22 (50%)	7 (70%)
L858R	28 (43%)	3 (27.2%)	22 (50%)	3 (30%)
G719X	1 (1.7%)	1 (9.2%)	0	0
**Line of EGFR TKIs, n (%)**				
First line	49 (75.3%)	6 (54.5%)	34 (77.2%)	9 (81.8%)
Later line	16 (24.7%)	5 (45.5%)	9 (22.8%)	2 (18.2%)
***EGFR* mutation subtypes, n (%)**				
Del19	36 (55.3%)	7 (63.6%)	22 (50%)	7 (70%)
L858R	28 (43%)	3 (27.2%)	22 (50%)	3 (30%)
Uncommon^a^	1 (1.7%)	1 (9.2%)	0	0
**Type of EGFR TKIs, n (%)**				
Gefitinib	38 (58.4%)	8 (72.7%)	26 (59%)	4 (40%)
Erlotinib	22 (33.8%)	2 (18.1%)	14 (31.8%)	6 (60%)
Afatinib	5 (7.8%)	1 (9.2%)	4 (9.2%)	0
Duration of TKI treatment, median (IQR, months)	9.3 (4.8–19.1)	2.8 (1.8–3.2)	9.6 (5.9–13.8)	31.1 (28.8–35.9)

*Del19* exon 19 deletion, *EGFR* epidermal growth factor receptor, *IQR* interquartile range.

To define underlined molecular characteristics according to the response of treatment, we explored 10 oncogenic signaling pathways which frequent genetic alterations and might be candidates of functional contributions including; cell cycle, Hippo, Myc, Notch, Nrf2, PI-3-Kinase/Akt, RTK-RAS, TGF-β, p53 and β-catenin/Wnt signaling^21^. A list of genes in each pathway and pattern of alterations are shown in additional information: Table S9, Fig. S10A-S10J. A tumor with one or more gene alterations, either mutation or CNAs in the pathway, was considered pathway alterations. The frequency of 10 pathway alterations was in range the of 18–84% (18.4% Nrf2, 27.6% TGF-β, 30% Myc, 52.3% β-catenin/Wnt, 56% Notch, 60% cell cycle, 76.9% RTK-RAS, 69% Hippo, 83% PI-3-Kinase/Akt, and 84% p53 pathway). In general, the prevalence of individual genetic alterations in our study were; *TP53* mutation 60%, *MDM2* copy number gain (CNG) 15%, *MET* CNG 30%, *ERBB2* CNG 7.6%, *PIK3CA* alteration 16%, *CDK4* CNG 16.9%, *CDK6* CNG 26.1%, *RB1* alteration 13.8%, *CCNE1* CNG 21%. The majority of frequency alterations, except *CDK6* and *CCNE1*, were similar to the *EGFR* mutation-positive NSCLC literature^22^.

**Figure 2:**
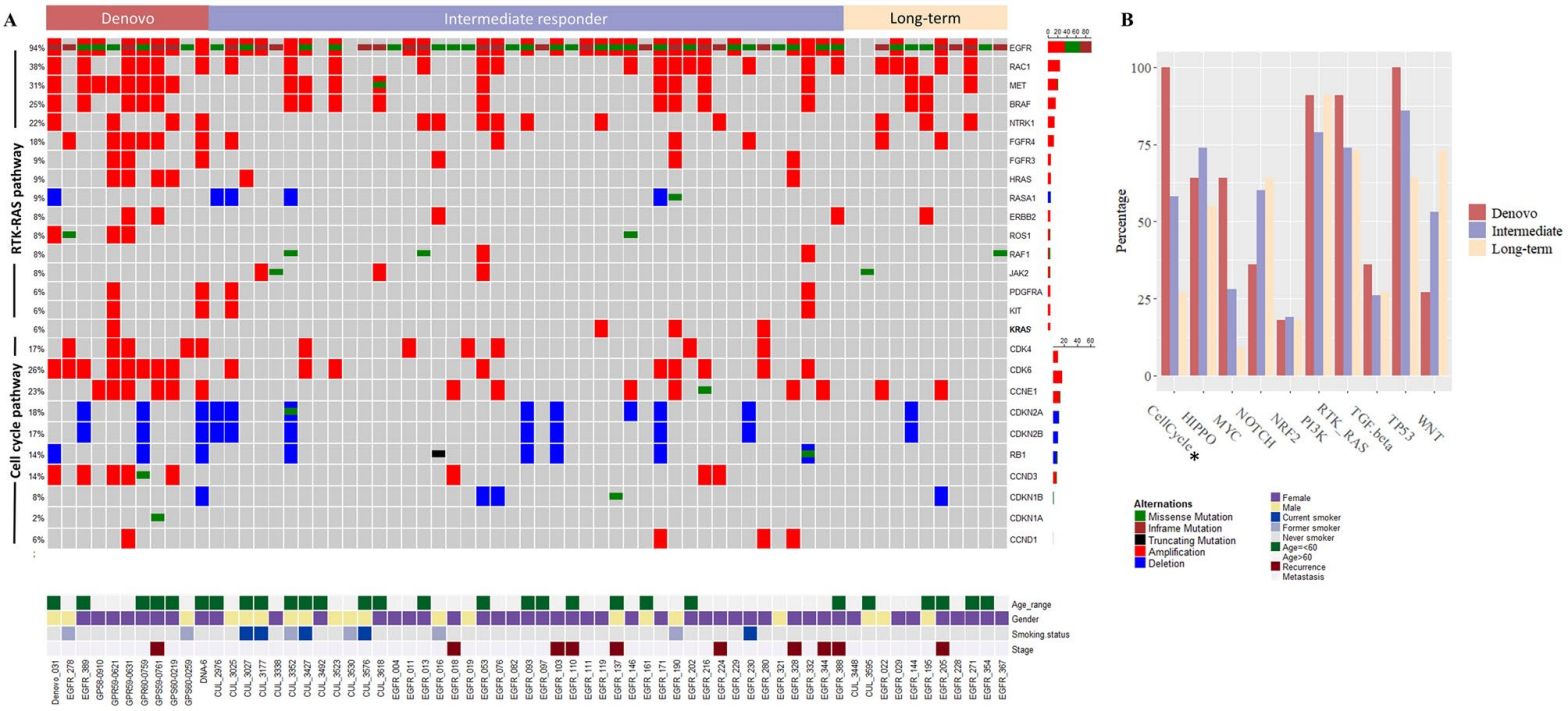
Individual details of patient factors with genomic alterations including SNVs and CNAs in RTK-RAS and cell cycle pathway (**A**). The cell cycle pathway was the only pathway that showed statistically significant (*q*-value 6 × 10^–4^) results among 10 oncogenic signaling pathways (**B**). The frequency of cell cycle genomic alterations was lowest, 27% in LTRs, 58% in IRs and enriched (100%) in de novo resistance.

**Figure 3:**
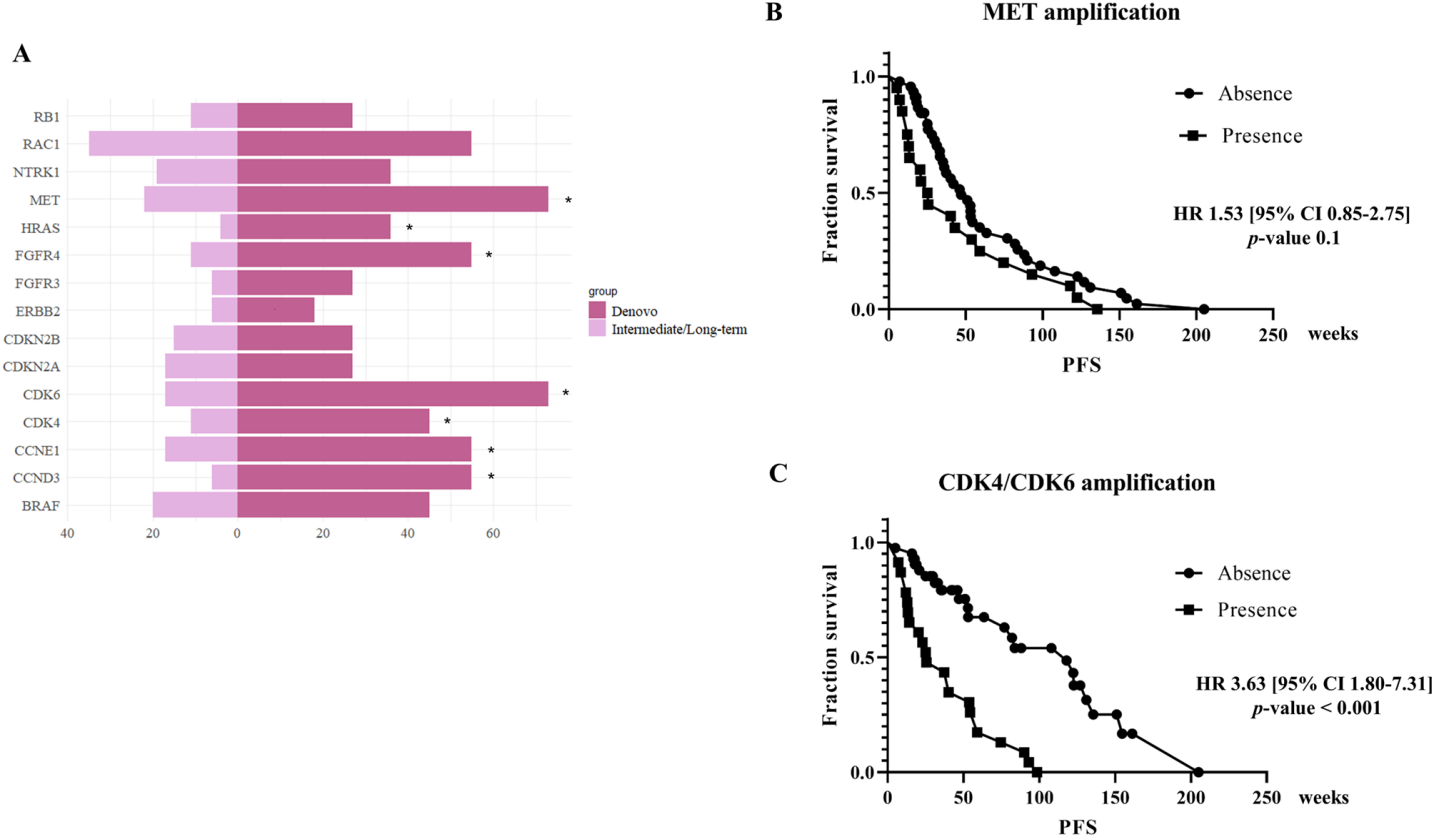
Comparison of 15 gene alteration frequencies among de novo resistance (n = 11) and IRs/LTRs (n = 54). We found a significant *q*-value < 0.05 in only 7 gene alterations; *CDK6*, *CCNE1, CDK4, CCND3, MET, FGFR4* and *HRAS* (including amplification and variant mutation) which enrich in de novo resistance [range 36–73%] compared to IRs/LTRs [range 4–22%] (**A**). Co-occurrence of MET amplification favors shorter EGFR TKI disease control than the absence of MET amplification with a median PFS 25 weeks vs. 47 weeks (HR 1.53 [95% CI 0.85–2.75, *p*-value 0.1] (**B**). While the presence of either *CDK4* or *CDK6* amplification significantly correlated with de novo resistance with the HR of PFS 0.63 [95% CI 1.80–7.31, *p*-value < 0.001] (**C**).

Cell cycle, RTK-RAS and PI-3-Kinase/Akt were the significant alteration pathways among treatment groups with the *p*-value of 6 × 10^–5^, 0.02 and 0.02, respectively. The *P*-value for significant pathways were adjusted by Benjamini–Hochberg Method which revealed significantly less than 0.05 in only cell cycle pathways (*q*-value 6 × 10^4^) (Fig. 2B). Individual genetic alterations per pathway were shown in Fig. 2A, Fig. S10A-S10J and additional information: Table S10. Either the amplification of *CDK4* or *CDK6* was found significant in the de novo resistance group, contrary to IRs and LTRs (91%, 27.9% and 0%, respectively). No correlation between significant alteration in cell cycle, RTK-RAS pathway and clinical phenotypes such as age, sex, smoking status, stage of disease was present. Among those significant pathways, we found a significant *q*-value < 0.05 in only 7 gene alterations; *CDK6*, *CCNE1, CDK4, CCND3, MET, FGFR4* and *HRAS* (including amplification and variant mutation) which enrich in de novo resistance [range 36–73%] compared to IRs/LTRs [range 4–22%] (Fig. 3A). Co-occurrence of MET amplification favors shorter EGFR TKI disease control than the absence of MET amplification with a median PFS of 25 weeks vs. 47 weeks (HR 1.53 [95% CI 0.85–2.75, *p*-value 0.1] (Fig. 3B). The discriminative effect was significant in the presence of either *CDK4* or *CDK6* amplification. Median PFS of EGFR TKI was 25 weeks compared to 118 weeks in the absence of those molecular markers with the HR of progression-free survival 3.63 [95% CI 1.80–7.31, *p*-value < 0.001] (Fig. 3C). For individual analysis, the presence of *CDK6* amplification was shown to shorten PFS of EGFR TKI with the HR of PFS 2.22 [95% CI 1.24–4.0, *p-*value 0.007]. The presence of *CDK4* amplification (16%), with less prevalence than *CDK6* amplification (26%), was also shown a trend of shortened PFS of EGFR TKI with the HR of PFS 1.79 [95% CI 0.92–3.48, *p*-value 0.08] (Fig S4). Furthermore, there was no *CDK4/CDK6* amplification in the long-term EGFR TKI responder. Median *CDK4* and *CDK6* integer copies number estimation by PureCN were 7 [range 2–244] and 5 [range 2–9]. The validation of *CDK4/6* amplification was conducted hybrid capture-based NGS OrigiMed Gene variation testing kit (ONCOSNAP pro)^23^ in 5 adequate specimens. The results were shown in additional information table S13. Consistent *CDK4* amplification in all but not for *CDK6* amplification was found. As OrigiMed Gene amplification threshold for amplification was over 6 copies. Five specimens which had *CDK6* amplification in range 2.9–6 using OrigiMed had been excluded by the algorithm. We analyzed the discriminative effect varying the WES threshold of amplification by using a calculated integer copy number. The HR of PFS were 1.92 [95%CI 1.09–3.41, *p*-value 0.02] and 2.17 [95% CI 1.13–4.15, *p*-value 0.01] for an amplification threshold of 4 and 6 respectively.

### Multivariate Cox regression analysis of PFS and OS including clinico-genomic characteristic

**Figure 4:**
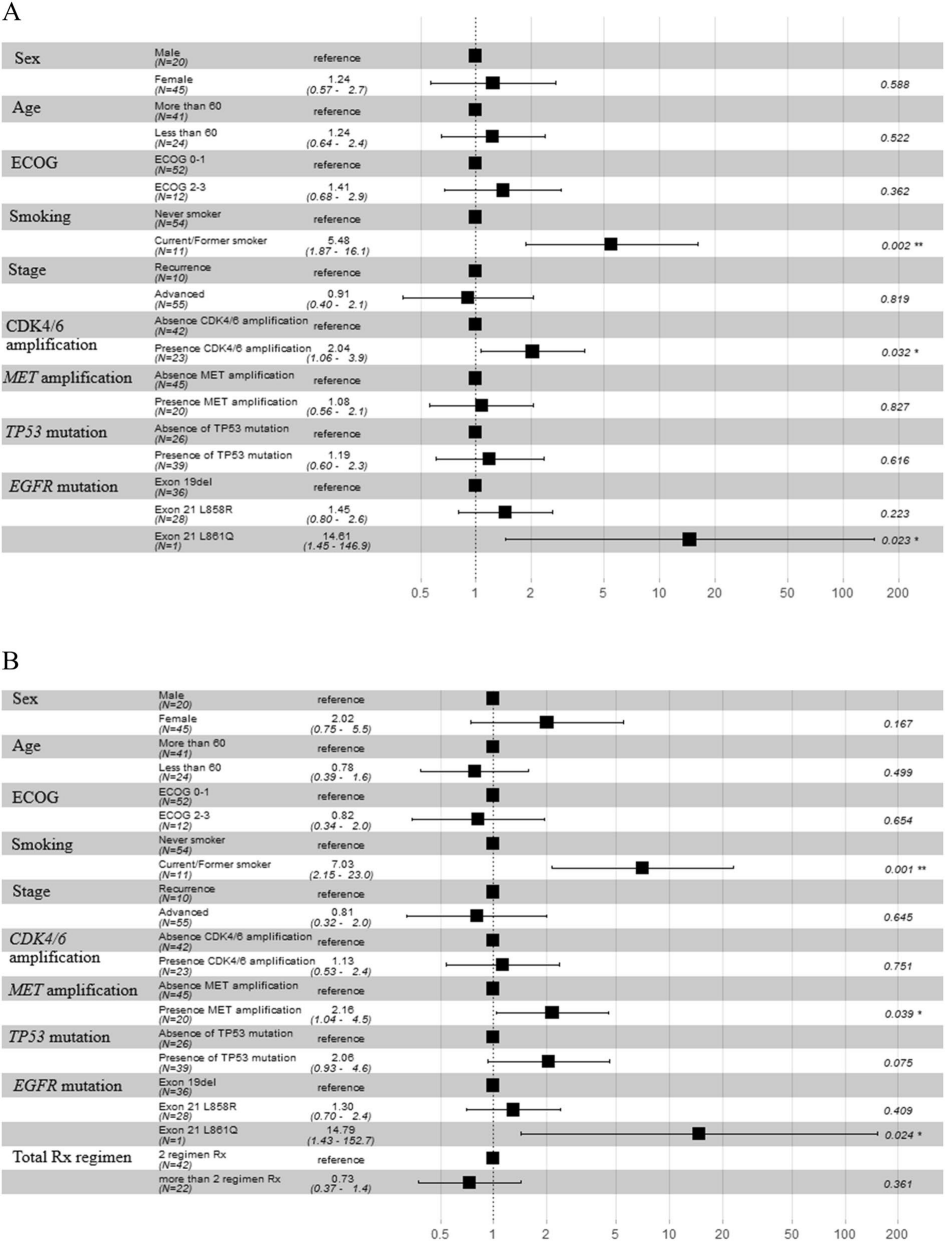
Multivariate analysis of PFS (**A**) and OS (**B**) in 65 advanced stage NSCLC who had adequate tissue for WES.

The median PFS and OS of 65 patients who had available FFPE to perform WES were 10 months [range 1.2–51.2] and 22.1 months [range 3.2–103.2] respectively. We conducted multivariate Cox regression analysis including sex, age, ECOG PS, smoking status, stage and molecular alterations including *CDK4/6* amplification, *MET* amplification, *TP53* mutation and *EGFR* mutation subtype. We found that uncommon *EGFR* mutation (L861Q) HR 14.61 [95% CI 1.45–146.9, *p*-value = 0.023], current/former smoking status HR 5.48 [95% CI 1.87–16.1, *p*-value = 0.002] and presence of *CDK4/6* amplification HR 2.04 [95%CI 1.06–3.9, *p*-value = 0.03] were significantly associated with shorten PFS (Fig. 4A). Regarding OS, we added total regimen of treatment into aforementioned multivariate Cox regression analysis. We found that uncommon *EGFR* mutation (L861Q) HR 14.79 [95% CI 1.43–152.7, *p*-value = 0.024], current/former smoking status HR 7.03 [95% CI 2.1–23.0, *p*-value = 0.001] were significantly associated with OS, consistent with PFS. Furthermore, presence of *MET* amplification HR 2.13 [95%CI 1.04–4.5, *p*-value = 0.03] and presence of *TP53* mutation HR 2.06 [95% CI 0.93–4.6, *p*-value = 0.07] were also associated with shorten OS (Fig. 4B).

## Discussion

We analyzed the clinicopathological and molecular features of a subset of patients that is intrinsically resistant to EGFR TKI treatment, although this subset represented only 8% of our study population. We found that uncommon *EGFR* mutation was an independent factor associated with de novo resistance compared to both IRs and LTRs. It is well known that uncommon *EGFR* mutations are a heterogeneous group with variable responses to EGFR TKIs, contrary to LTRs which represented 19% of our study. The median duration of EGFR TKIs treatment in this group was 32.4 months. LTRs showed a substantially lower number of metastatic sites (*p*-value 0.002) and almost exclusively oligo-progression. Patients with recurrent disease who displayed favorable responses to EGFR TKI treatment may partly be explained by their small disease burden and low tumor heterogeneity^24,25^. Additionally, a meta-analysis showed that never smokers had better PFS benefits than ever smokers in patients who harbored the activating *EGFR* mutation and received EGFR TKIs^26^. However, we found that this factor was not associated with the outcome of disease control.

Besides specific clinical factors, we found that diverse genomic landscapes underlined distinct EGFR TKI responses. Varying mechanisms of de novo resistance in sensitizing *EGFR* mutation were reported such as de novo co-occurrence of *PIK3CA*^27^, *PI3K*/*AKT*/*mTOR*^28^, *PTEN* loss^29^, *MET* alteration^30–32^ and *TP53* mutation^33^. Here, we focused on 10 frequent oncogenic signaling pathways; cell cycle, Hippo, Myc, Notch, Nrf2, PI-3-Kinase/Akt, RTK-RAS, TGF-β, p53 and β-catenin/Wnt signaling which were previously shown to be significant among various cancer types, involving tumorigenesis, cell proliferation, metastasis and angiogenesis^34,35^. Targeting signaling pathways has been a challenge in defining a novel cancer treatment. Among them, RTK-RAS and cell cycle pathways were the most frequent alterations in no-mutation selected adenocarcinoma of the lung with a frequency of 74% and 56%, respectively^21^. These frequencies of pathway alterations were consistent with our *EGFR* mutation-positive study; 77% in RTK-RAS and 60% in cell cycle pathway, respectively. We found that cell cycle pathway alteration was the only significant pathway alteration (*q*-value < 0.05) with essential frequency in de novo compared to IRs and LTRs (100% vs. 58% vs. 27%, respectively). *CCNE1, CDK4/6* and *CCND3* were major contributors of cell cycle pathway alteration with *q*-value < 0.05. Altered cell cycle expression has also been correlated with acquired EGFR TKI resistance^36^. Broadened exploration of gene alteration in our study confirmed prior cfDNA targeted sequencing of 68 genes that revealed significant cell cycle pathways and the presence of *CDK4/6* alterations, which were significantly associated with non-responder of osimertinib^37^.

Furthermore, *MET* alteration was also enriched in de novo resistance with *q*-value < 0.05. Despite varying techniques and definitions used to define *MET* amplification^38^, it is well-known as an important role in de novo and acquired EGFR TKI resistance through bypass activating ERBB/PI3K-Akt signaling pathway^30,39–42^. The presence of *MET* amplification was significantly associated with shortened OS in multivariate Cox regression analysis with the HR of 2.13 [95% CI 1.04–4.5, *p*-value = 0.03] (Fig. 4B). Missense *TP53* mutation, which has previously shown the predictive impact of EGFR TKI treatment in meta-analysis^33^, had a higher prevalence in de novo EGFR TKI than IRs and LTRs (81% vs. 60% vs. 36%, respectively). The prevalence of co-occurrence alteration of *TP53* and *CDK4/6* amplification was 24.6% (81% in de novo EGFR TKI, 16% in IRs and none in LTR). The presence of missense *TP53* mutation was shown as potential prognostic significance to OS but not for PFS in multivariate Cox regression analysis with the HR of OS 2.06 [95% CI 0.93–4.6, *p*-value = 0.07] (Fig. 4). Our results were consistent with previous publication^43^. The prevalence of co-alteration *RB1* alteration and *CDK4/6* amplification in *EGFR* mutation NSCLC patient was 7.6% (de novo 27%, IRs 4% and none in LTR). *RB1* alteration was correlated poor prognostic outcome in non-select genomic subgroup advanced NSCLC^44^ but not in *EGFR*-selected population. Univariate analysis in *RB1* alteration was not shown significant in term of PFS and OS (HR of PFS 1.76 [95% CI 0.85–3.64, *p*-value 0.1], HR of OS 1.78 [95% CI 0.78–4.02, *p*-value 0.1]. Furthermore, non-canonical *CDK4/6* substrates such as transcription factor Forkhead Box 1 (*FOXM1*), certain glycolytic enzymes and nuclear factor of activated T cell (*NFAT*) family members, let activity of *CDK4/6* even lack of *RB1* function^45–48^. The presence of either *CDK4* or *CDK6* amplification in the pretreatment specimen served as a predictive biomarker for EGFR TKI resistance in sensitizing *EGFR* mutation. Correlation with calculated integer copy number, using *CDK4/6* amplification threshold either 2, 4, 6 has discriminate predictive significance to EGFR TKI. Combination EGFR TKI treatment plus anti-CDK4/6 inhibitors are possible to overcome de novo EGFR TKI clonal resistance. Dual *CDK4/6* and *EGFR* blockage shown in vitro activity to prevent or delay resistance in *EGFR* mutant NSCLC^49^.

Although we focused on significant pathway alteration in whole exome sequencing, our study has some limitations. First, there was a lack of fusion alteration in our analysis. Fusion alteration has been reported as an uncommon mechanism of acquired EGFR TKI resistance^50–53^. Nevertheless, co-occurrence fusion in pretreatment *EGFR* mutation-positive was reported at a low frequency (0.9%)^52^. Second, we used cohort-normal workflow, which is required in silico prediction by using allele-specific copy number to calculate the posterior probability to define the variant as somatic variant status (See “Methods”). Using an exploratory cohort of 65 tumor-normal pair resectable adenocarcinoma of lung, revealed that 84% of all non-synonymous mutations in “cohort-normal” concordance to 61.3% of “match-normal” workflow. Subclonal mutations which had low allelic fractions or low purity might be the reason for lower precision accuracy. Nevertheless, we selected high concordance, 92.3% in a limited 206 genes from 10 significant pathway analyses, which cover all significant co-occurrence alterations. Third, our average depth coverage of WES was 65× [range 40–94×] was significantly different among the EGFR TKI treatment group which might impact the detection number of low allelic fraction mutation. However, this sequencing coverage depth is still enough to define a significant pathway and genomic alteration that correlates with de novo resistance. Fourth, the copy number threshold to define amplification was adjusted (> 0.3), more precisely than the pipeline recommendation. The gene-level segment integer copy number was parallel conducted using PureCN. The algorithm was previously shown good concordance with absolute copy number by targeted NGS- Foundation Medicine platform^54^. However, currently hybrid capture-based NGS has diverse thresholds for amplification. The threshold used in FoundationOne® Heme for identifying a copy number amplification is 5 for *ERBB2* and 6 for all other genes while the threshold used in OrigiMed is 6 for all. Lastly, we didn’t assess prognostic significance for non-significant genes such as *AURKB* (1.5%) and *RBM10* (6%), even reported the prognostic significance associated with EGFR TKI. Four of 65 discrepancies of EGFR mutation results between WES and Cobas® mutation testing were found. Integrative Genomics Viewer analysis (IGV) on the bam file was performed; all specimens had EGFR exon 19 deletion reads less than 15 which were removed by our algorithm.

## Materials and methods

### Study population

All methods were carried out in accordance with the declarations of Helsinki. The Institutional Review Board (IRB), Faculty of Medicine, Chulalongkorn University approved the study protocol (IRB 298/60). Written Informed consent was waived from individual study participants according to the ethics committee/IRB, Faculty of Medicine, Chulalongkorn University policy for retrospective study. The permission to conduct the study was provided by the director of the hospital. We retrospectively analyzed patients with pathologically confirmed recurrence or metastatic NSCLC diagnosed between 2011 and 2018 at King Chulalongkorn Memorial Hospital (KCMH). *EGFR* mutation testing was determined by cobas® *EGFR* Mutation Test v2 kit. Patients with NSCLC harboring activating *EGFR* mutations who received 1st or 2nd generation EGFR TKIs were included in our study, excluding osimertinib according to limited participants (n = 9) (Fig. 1). All patients were assessed for tumor responses and followed up every two to three months as the standard protocol of our institution. Objective response rate (ORR) was determined according to the Response Evaluation Criteria in Solid Tumors version 1.1 (RECIST v1.1) and classified as a progressive disease (PD), complete response (CR), partial response (PR), or stable disease (SD). Patients were categorized into three groups based on responsiveness to EGFR TKI treatment: (1) those with de novo EGFR TKI resistance who were defined as the best response were PD or SD less than 3 months while receiving EGFR TKI^16^ (de novo resistance); (2) those who developed acquired resistance to EGFR TKIs according to the proposed criteria by Jackman^17^ (Intermediate responder) and (3) those treated with EGFR TKIs for at least 2 years^18^ (Long-term responder). Independent radiologist blind to molecular characteristic had reviewed imaging responses of 65 patients who obtained available tissue for WES.

### Exome sequencing analysis

Genomic DNA was extracted from paraffin-embedded tissue, using Qiagen FFPE DNA extraction kits following manufacturer protocol. We used leftover extracted genomic DNA after cobas® *EGFR* Mutation Test as part of standard testing in advanced stage disease. After performing quality control (QC), qualified samples were proceeded to library construction. The genomic library was constructed with SureSelectXT V6 + UTR library prep kit (Illumina, San Diego, CA, USA) and was sequenced using NovoSeq to generate 150 bp paired-end reads at Macrogen Inc. (Seoul, Korea). The analytical pipeline of “cohort-normal” which showed high concordance rate (*R*^2^ 0.99) to “match-normal” workflow in 10 significant pathways (206 genes) was explored in our study. The analysis of “cohort-normal” workflow was compared with our “match-normal” workflow, using 65 pair tumor-normal fresh tissue WES from resectable lung cancer patients who had received surgical procedures at The King Chulalongkorn Memorial Hospital. Written Informed consent was obtained in all resectable lung cancer participants. We selected those 65 specimens as retrospective manner based on EGFR TKI response with an adequate amount of specimen for WES, enriched in de novo EGFR TKI resistance. Ninety-eight percent of the second cohort WES had sensitizing mutation; composed of 55% EGFR exon 19 deletion, 43% exon 19 L858R, and one patient with exon 21 L861Q mutation. Sixty-five WES advanced stage NSCLC were categorized to 11 de novo resistance, 43 intermediate responders and 11 long-term responders. Pooled normal in the “cohort-normal” pipeline was obtained from either 65 normal lung tissue or leucocytes from the first exploratory cohort. Preprocessing steps and filtering for variant and copy number alteration (CNAs) discovery are described in the supplementary information. 

### Statistical analysis

The correlation of all categorical variables was analyzed using Kruskal–Wallis test. Significant correlation of two categorical variables was analyzed using two-sided Fisher’s exact test or Chi-squared test for *p*-value calculations, while correlation of two continuous variables was conducted using Wilcoxon rank-sum test. FDR *p*-values were calculated by Benjamini–Hochberg Method from all correlation *p*-values in this cohort. Progression-free survival (PFS) was calculated from the first day of treatment with EGFR TKI to disease progression or death. Overall survival (OS) was calculated from the date of diagnosis of recurrent or metastatic disease until the date of death. Patients were censored on June 30, 2020, if alive. Survival analysis was performed with a Kaplan–Meier analysis and log-rank test. Multivariate analysis was performed by binary logistic regression or Cox’s proportional hazards regression model when appropriate. The level of statistical significance was determined as a *p*-value less than 0.05. All statistical analyses were conducted using SPSS 23.0 (SPSS Inc, Chicago, Illinois, USA) and R package version 3.6.3.

## Supplementary Information

Supplementary Information 1.

Supplementary Information 2.
