# Supplementary material for: Co-occurrence CDK4/6 amplification serves as biomarkers of de novo EGFR TKI resistance in sensitizing EGFR mutation non-small cell lung cancer
Source: Sci Rep. 2022 Feb 9;12:2167. doi: 10.1038/s41598-022-06239-y (PMC8828869; doi:10.1038/s41598-022-06239-y)

**Supplementary information**

**Methods: Preprocessing step for variant and CNAs discovery**

We analyzed data with target sequences of approximately 90 Mb. Unmapped BAM was generated from Fastq raw data, aligned with hg19 reference using BWA version 0.7.17 and processed by using GATK best practice pipeline though Genome Analysis Toolkit recommendation (GATK version 4.1.0.0) (1) including MarkDuplicates, base quality score recalibration, indel realignment, duplicated removal. High confidence somatic substitutions were identified using MuTect2 algorithm which has shown the ability to detect low allele fractions (2). Estimate cross-sample contamination using GetPileupSummaries and estimate sample actifact using CollectSequencingArtifactMetrics and filtering for confident somatic calls using FilterMutectCalls and FilterByOrientationBias. All variants were annotated using Oncotator v.1.9.9.0.

Upon identifying the significant variant which has been filtered by Mutect2, variants at positions with total read depth of less than 15, variant allele frequency of less than 0.03 and more than 0.97, were disregarded. Further filtering of “match-normal” pipeline including putative common germline variant alleles those present in MAF >1% from population variant databases (PVDs) including1000 Genomes Project (2015 Jan. <http://www.1000genomes.org/>), ESP6500 database (<http://evs.gs.washington.edu/EVS/>) and ExAC (<http://exac.broadinstitute.org/about>). Additional filtering step of “cohort-normal” pipeline included variant presence of at least one in 65 pool normal references. Retained variants were defined as oncogenic by OncoKB version 2.7 (3) or predicted as somatic status by PureCN 1.21.0. (4). PureCN algorithm using posterior probability cutoff of > 0.8 and < 0.2 for somatic and germline variants, respectively. We used 2 subsets of significance for further analysis. The first set was the 307 significant mutated genes from lung adenocarcinoma (TCGA, Firehose Legacy) and 6 publications (5-10) which composed of 2,678 LUAD patients analyzed by MutSigCV version 1.3.1. (*q* < 0.1 and nonsilent mutation n > 5) (11) (additional information: Table S6). The second set was 206 significant genes from 10 significant pathway analyses which showed significant alteration among 33 cancer types (9). Mutated genes were defined if at least one non-synonymous mutation was found in the coding sequence of that gene. We compared concordance non-synonymous variants of “match-normal” and “cohort-normal” in all and significant mutated genes.

Copy number alteration (CNAs) were analysed using GATK somatic CNVs version 4.1.0.0. Panel of normal was created running the initial coverage collection tools individually on 65 normal WES and combining copy ratio data by CreateReadCountPanelOfNormals. Depth of coverage at capture targets in tumor samples were normalized by read coverage spanning a target segment with the total number of aligned reads and proportionally calibrated to estimate the copy ratio using depths observed in a panel of normal. The GATK somatic CNVs pipeline included PreprecessIntervals, CollectFragmentCounts, ModelSegments and CallCopyRatioSegments with lower and upper bounder of log2 neural copy segment ratio at -0.3 and 0.3 to classify amplified and deleted segments. The preprocessing and variant discovery pipeline was run through WDL workflow on Cromwell execution engine platform [doi: 10.7490/f1000research.1114631.1]. Tumor ploidy and purity estimation were conducted using PureCN which previously showed high concordance in tumor-only WES workflow compared to SNP6 microarray and match-normal WES workflow (12). Gene-level segment integer copy number estimation was conducted by PureCN. This algorithm was previously shown good concordance of absolute copy number adjusted for purity and ploidy with Foundation Medicine (4).

**Progression-free survival analysis**

Patients were categorized into 3 groups; de novo resistance (N = 28, 8%) with a median PFS of 2.1 months (95% CI, 1.3 – 2.8 months), intermediate responders (IRs, N = 240, 72%) with a median PFS of 11.0 months (95% CI, 9.9 – 12.1 months) and long-term responders (LTRs, N = 64, 19%) with a median PFS of 34.9 months (95% CI, 32.0 – 37.7 months) (Supplementary Fig. S1A). Multivariate Cox’ regression analysis according to progression-free survival was conducted. Hazard ratio (HR) of worse ECOG performance status (>2 vs. 0-1) was 1.71 [95% CI: 1.71-2.49], *p*-value 0.006). While HR of advanced stage disease at diagnosis and uncommon *EGFR* mutation subtype were 1.78 [95% CI: 1.28-2.47], *p*-value 0.001 and 1.84 [95% CI: 1.13-3.0], *p*-value 0.01, respectively (Supplementary Table S2). Furthermore, we found that a high number of metastatic sites were also associated with shorter PFS from EGFR TKI (> 3 vs. 1-2 sites, HR 1.79 [95% CI 1.31-2.44], *p*-value < 0.001). No differences in PFS outcome were identified across patient subgroups with respect to age, gender, smoking status, histology, baseline liver or brain metastasis, the treatment lines and generation of TKI.

**Overall survival analysis**

The median follow-up duration was 51.5 months (95% CI, 45.9 – 57.0). A total of 217 patients (65%) were ceased. The median OS of the overall study cohort was 32.9 months (95% CI, 27.9 – 37.8 months). Patients with *de novo* resistance had a significantly shorter OS than patients with IRs and LTRs, respectively. (median OS was 7.2 months [95% CI 3.5 – 10.9], 25.7 months [95% CI 22.1 – 29.3], and 80.5 months [95% CI 65.2 – 95.8], respectively; *p*-value <0.001) (Figure S2). A multivariate Cox’s regression model showed ECOG PS, number of metastatic sites, baseline liver metastasis, the treatment lines of TKI, subsequent osimertinib treatment and the response to EGFR TKIs remained as independent prognostic factors for OS (Supplementary Table S3).

**Subsequent therapies**

At the data cut-off point, 26 patients (8%) were still receiving EGFR TKI treatment. 306 patients discontinued EGFR TKIs, the majority of whom (95%) discontinued due to disease progression. Of these, 159 patients (48%) received subsequent anticancer therapies. 27%, 12% and 9% had received 1, 2 or at least 3 lines of subsequent therapy. This included 125 patients (38%) who received EGFR TKI beyond progression. 11 *de novo* resistance patients received at least one-line of subsequence systemic therapy, 1 patient received two lines, 1 patient received three or more lines. 68 IR patients received one further line of systemic therapy, 33 patients received two lines, 23 patients received three or more lines. 12 LTR patients received one further line of systemic therapy, 5 patients received two lines, 5 patients received three or more lines. 20 of 64 LTRs (31%) were still receiving EGFR TKIs treatment at the time of analysis, so they have yet to receive any subsequent treatment.

162 patients (49%) who progressed after EGFR TKIs treatment were further tested for secondary T790M mutation and 84 patients (52%) were found to have the T790M mutation. Of these, 86 patients received osimertinib, a 3rd generation of EGFR TKIs, as subsequent treatment (1 patient had *de novo* T790M and 1 patient had T790M negative received osimertinib). Details of treatment after progression on TKI are provided in Supplementary Table S4.

According to Gardara et al (13), among subtype of progressive disease after EGFR TKIs resistance, 170 patients were classified as systemic progression (51%), 44 patients as isolated CNS progression (13%) and 72 patients as oligo-progression (22%). Interestingly, LTR patients were more likely to develop oligo-progression (41%) than *de novo* resistance (18%) and IRs (17%), respectively (p<0.001) (Supplementary Table S5). Additionally, patients who developed oligo-progression had a significantly longer OS than patients who developed CNS progression and systemic progression, respectively. (median OS was 42.3 months [95% CI 27.4 – 57.3], 29.4 months [95% CI 21.7 – 37.0], and 27.0 months [95% CI 20.2 – 33.8], respectively; p = 0.02). (Supplementary Table S3)

**Reference;**

1. DePristo MA, Banks E, Poplin R, Garimella KV, Maguire JR, Hartl C, et al. A framework for variation discovery and genotyping using next-generation DNA sequencing data. Nat Genet. 2011;43(5):491-8.

2. Cibulskis K, Lawrence MS, Carter SL, Sivachenko A, Jaffe D, Sougnez C, et al. Sensitive detection of somatic point mutations in impure and heterogeneous cancer samples. Nat Biotechnol. 2013;31(3):213-9.

3. Chakravarty D, Gao J, Phillips SM, Kundra R, Zhang H, Wang J, et al. OncoKB: A Precision Oncology Knowledge Base. JCO Precis Oncol. 2017;2017.

4. Riester M, Singh AP, Brannon AR, Yu K, Campbell CD, Chiang DY, et al. PureCN: copy number calling and SNV classification using targeted short read sequencing. Source Code Biol Med. 2016;11:13.

5. Cancer Genome Atlas Research N. Comprehensive molecular profiling of lung adenocarcinoma. Nature. 2014;511(7511):543-50.

6. Ding L, Getz G, Wheeler DA, Mardis ER, McLellan MD, Cibulskis K, et al. Somatic mutations affect key pathways in lung adenocarcinoma. Nature. 2008;455(7216):1069-75.

7. Govindan R, Ding L, Griffith M, Subramanian J, Dees ND, Kanchi KL, et al. Genomic landscape of non-small cell lung cancer in smokers and never-smokers. Cell. 2012;150(6):1121-34.

8. Jordan EJ, Kim HR, Arcila ME, Barron D, Chakravarty D, Gao J, et al. Prospective Comprehensive Molecular Characterization of Lung Adenocarcinomas for Efficient Patient Matching to Approved and Emerging Therapies. Cancer Discov. 2017;7(6):596-609.

9. Sanchez-Vega F, Mina M, Armenia J, Chatila WK, Luna A, La KC, et al. Oncogenic Signaling Pathways in The Cancer Genome Atlas. Cell. 2018;173(2):321-37 e10.

10. Rizvi NA, Hellmann MD, Snyder A, Kvistborg P, Makarov V, Havel JJ, et al. Cancer immunology. Mutational landscape determines sensitivity to PD-1 blockade in non-small cell lung cancer. Science. 2015;348(6230):124-8.

11. Lawrence MS, Stojanov P, Polak P, Kryukov GV, Cibulskis K, Sivachenko A, et al. Mutational heterogeneity in cancer and the search for new cancer-associated genes. Nature. 2013;499(7457):214-8.

12. Oh S, Geistlinger L, Ramos M, Morgan M, Waldron L, Riester M. Reliable Analysis of Clinical Tumor-Only Whole-Exome Sequencing Data. JCO Clin Cancer Inform. 2020;4:321-35.

13. Gandara DR, Li T, Lara PN, Kelly K, Riess JW, Redman MW, et al. Acquired resistance to targeted therapies against oncogene-driven non-small-cell lung cancer: approach to subtyping progressive disease and clinical implications. Clin Lung Cancer. 2014;15(1):1-6.

**Table S1.** Univariate and multivariate logistic regression analyses of clinical variables of EGFR TKI long-term responder (LTRs) vs. intermediate-responder (IRs)

| **LTRs versus IRs**  **Variables^a^** | **Univariate** | | **Multivariate** | |
| --- | --- | --- | --- | --- |
|  | **OR (95% CI)** | **P value** | **OR (95% CI)** | **P value** |
| Age (<60/>60) | 0.80 (0.45 – 1.42) | 0.44 |  |  |
| Sex (male/female) | 0.81 (0.45 – 1.47) | 0.50 |  |  |
| ECOG PS (>2/0-1) | 0.47 (0.16 – 1.39) | 0.17 |  |  |
| Smoking (current-former/never) | 1.01 (0.47 – 2.18) | 0.98 |  |  |
| Histology (non-ADC/ADC) | 0.99 (0.27 – 3.61) | 0.98 |  |  |
| Stage at diagnosis (M1/M0) | 0.53 (0.29 – 0.96) | **0.04*** | 0.97 (0.35 – 2.73) | 0.96 |
| Curative surgery (no/yes) | 0.51 (0.28 – 0.94) | **0.03*** | 0.54 (0.19 – 1.57) | 0.26 |
| Number of metastatic sites (>3/1-2) | 0.20 (0.08 – 0.52) | **0.001*** | 0.20 (0.08 – 0.53) | **0.001*** |
| Brain metastasis (yes/no) | 0.64 (0.30 – 1.34) | 0.23 |  |  |
| Liver metastasis (yes/no) | 0.75 (0.30 – 1.90) | 0.55 |  |  |
| *EGFR* subtype (others/common) | 0.69 (0.19 – 2.44) | 0.56 |  |  |
| Common *EGFR* subtype (Del19/L858R) | 0.92 (0.52 – 1.62) | 0.76 |  |  |
| Line of TKI (first/later) | 1.71 (0.91 – 3.18) | 0.09 | 1.88 (0.98 – 3.57) | 0.06 |
| Generation of TKI (first/second) | Not estimated | 1 |  |  |
| First generation of TKI (gefitinib/erlotinib) | 0.70 (0.40 – 1.23) | 0.21 |  |  |

^a^Category after the slash (/) was set as reference category. ADC: adenocarcinoma, Del19: exon 19 deletion, ECOG PS: Eastern Cooperative Oncology Group performance status, EGFR: epidermal growth factor receptor, IRs: intermediate responders, LTRs: long-term responders, M0: recurrent disease, M1: metastatic disease, TKI: tyrosine kinase inhibitor.

**Table S2.** Cox’s proportional hazards regression model revealed factors associated with PFS of EGFR TKI treatment

| **Variables^a^** | **Univariate** | | **Multivariate** | |
| --- | --- | --- | --- | --- |
|  | **HR (95% CI)** | **P value** | **HR (95% CI)** | **P value** |
| Age (<60/>60) | 1.28 (1.02 – 1.62) | **0.04*** | 1.20 (0.92 – 1.56) | 0.18 |
| Sex (male/female) | 1.28 (1.01 – 1.62) | **0.05*** | 1.18 (0.86 – 1.63) | 0.31 |
| ECOG PS (>2/0-1) | 1.57 (1.12 – 2.21) | **0.009*** | 1.71 (1.17 – 2.49) | **0.006*** |
| Smoking (current-former/never) | 1.38 (1.02 – 1.87) | **0.04*** | 1.18 (0.81 – 1.73) | 0.39 |
| Histology (non-ADC/ADC) | 1.14 (0.67 – 1.96) | 0.63 |  |  |
| Stage at diagnosis (M1/M0) | 1.78 (1.34 – 2.38) | **<0.001*** | 1.78 (1.28 – 2.47) | **0.001*** |
| Curative surgery (no/yes) | 1.53 (1.14 – 2.05) | **0.005*** | 0.88 (0.50 – 1.55) | 0.66 |
| Number of metastatic sites (>3/1-2) | 2.02 (1.55 – 2.64) | **<0.001*** | 1.79 (1.31 – 2.44) | **<0.001*** |
| Brain metastasis (yes/no) | 1.22 (0.92 – 1.61) | 0.18 |  |  |
| Liver metastasis (yes/no) | 1.42 (0.98 – 2.06) | 0.06 | 1.11 (0.72 – 1.70) | 0.64 |
| *EGFR* subtype (others/common) | 1.42 (0.94 – 2.14) | 0.10 | 1.84 (1.13 – 3.00) | **0.01*** |
| Common *EGFR* subtype (Del19/L858R) | 1.05 (0.82 – 1.34) | 0.70 |  |  |
| Line of TKI (first/later) | 0.86 (0.68 – 1.09) | 0.21 |  |  |
| Generation of TKI (first/second) | 0.61 (0.34 – 1.09) | 0.09 | 0.62 (0.33 – 1.19) | 0.15 |
| First generation of TKI (gefitinib/erlotinib) | 1.02 (0.80 – 1.30) | 0.89 |  |  |

^a^Category after the slash (/) was set as reference category. ADC: adenocarcinoma, Del19: exon 19 deletion, ECOG PS: Eastern Cooperative Oncology Group performance status, EGFR: epidermal growth factor receptor, M0: recurrent disease, M1: metastatic disease, TKI: tyrosine kinase inhibitor.

**Table S3.** Univariate and multivariate Cox’s regression analysis according to overall survival

| **Variables^a^** | **Univariate** | | **Multivariate** | |
| --- | --- | --- | --- | --- |
|  | **HR (95% CI)** | **P value** | **HR (95% CI)** | **P value** |
| Age (<60/>60) | 0.96 (0.74 – 1.26) | 0.79 |  |  |
| Sex (male/female) | 1.17 (0.89 – 1.54) | 0.26 |  |  |
| ECOG PS (>2/0-1) | 2.63 (1.83 – 3.79) | **<0.001*** | 3.25 (1.48 – 7.16) | **0.003*** |
| Smoking (current-former/never) | 1.55 (1.10 – 2.20) | **0.01*** | 1.43 (0.76 – 2.68) | 0.26 |
| Histology (non-ADC/ADC) | 1.06 (0.56 – 2.00) | 0.86 |  |  |
| Stage at diagnosis (M1/M0) | 1.66 (1.18 – 2.31) | **0.003*** | 1.59 (0.55 – 4.59) | 0.39 |
| Curative surgery (no/yes) | 1.69 (1.20 – 2.39) | **0.003*** | 0.89 (0.32 – 2.49) | 0.82 |
| Number of metastatic sites (>3/1-2) | 1.88 (1.40 – 2.51) | **<0.001*** | 2.09 (1.17 – 3.73) | **0.01*** |
| Brain metastasis (yes/no) | 1.27 (0.92 – 1.75) | 0.15 |  |  |
| Liver metastasis (yes/no) | 1.77 (1.20 – 2.60) | **0.004*** | 2.47 (1.20 – 5.07) | **0.01*** |
| *EGFR* subtype (others/common) | 1.28 (0.82 – 2.01) | 0.28 |  |  |
| Common *EGFR* subtype (Del19/L858R) | 0.88 (0.66 – 1.16) | 0.36 |  |  |
| Line of TKI (first/later) | 1.48 (1.12 – 1.97) | **0.007*** | 3.40 (1.82 – 6.36) | **<0.001*** |
| Generation of TKI (first/second) | 0.58 (0.31 – 1.10) | 0.09 | 0.36 (0.12 – 1.09) | 0.07 |
| First generation of TKI (gefitinib/erlotinib) | 1.14 (0.86 – 1.51) | 0.36 |  |  |
| Pattern of PD  CNS PD/systemic PD  Oligo-PD/systemic PD | 1.03 (0.70 – 1.52)  0.62 (0.44 – 0.89) | 0.89  **0.009*** | 1.94 (0.94 – 4.00)  0.48 (0.24 – 0.93) | 0.07  **0.03*** |
| Treatment beyond PD (yes/no) | 0.64 (0.48 – 0.86) | **0.003*** | 1.70 (0.93 – 3.10) | 0.09 |
| Subsequent 3^rd^generation TKI (no/yes)^b^ | 3.29 (2.14 – 5.04) | **<0.001*** | 5.62 (3.08 – 10.23) | **<0.001*** |
| Number of systemic therapy (1-2/>3) | 1.55 (1.18 – 2.06) | **0.002*** | 1.31 (0.71 – 2.39) | 0.39 |
| EGFR TKIs responders  (De novo/LTRs)  (IRs/LTRs) | 31.75 (16.53 – 60.97)  6.18 (3.72 – 10.27) | **<0.001***  **<0.001*** | 51.03 (11.88 – 219.18)  8.30 (3.54 – 19.43) | **<0.001***  **<0.001*** |

^a^Category after the slash (/) was set as reference category. ADC: adenocarcinoma, Del19: exon 19 deletion, ECOG PS: Eastern Cooperative Oncology Group performance status, EGFR: epidermal growth factor receptor, IRs: intermediate responders, LTRs: long-term responders, M0: recurrent disease, M1: metastatic disease, PD: progressive disease, TKI: tyrosine kinase inhibitor.

^b^Only 162 patients who progressed after EGFR-TKIs treatment were further tested for secondary T790M mutation

**Table S4.** Subsequent treatment after progression on EGFR TKI

| **Characteristics** | **All**  **(N = 332)** | **De novo resistance**  **(N = 28)** | **Intermediate responders**  **(N = 240)** | **Long-term responders**  **(N = 64)** | ***P* values** |
| --- | --- | --- | --- | --- | --- |
| Number of systemic therapies, n (%)  1-2 regimens  >3 regimens | 214 (64.5%)  118 (35.5%) | 22 (78.6%)  6 (21.4%) | 143 (59.6%)  97 (40.4%) | 49 (76.6%)  15 (23.4%) | **0.01*** |
| Number of subsequent therapies, n (%)  0  1-2  >3 | 173 (52.1%)  130 (39.1%)  29 (8.7%) | 15 (53.6%)  12 (42.9%)  1 (3.6%) | 116 (48.3%)  101 (42.1%)  23 (9.6%) | 42 (65.6%)  17 (26.6%)  5 (7.9%) | 0.51 |
| Ongoing TKI treatment, n (%) | 26 (7.8%) | 0 | 6 (2.5%) | 20 (31.3%) | **<0.001*** |
| Treatment beyond progression, n (%) | 125 (37.7%) | 9 (32.1%) | 78 (32.5%) | 38 (59.4%) | **<0.001*** |
| Secondary T790M testing, n (%) | 162 (48.8%) | 5 (17.9%) | 123 (51.3%) | 34 (53.1%)) | **0.003*** |
| Secondary T790M positive, n (%) | 84 (51.9%) | 0 | 64 (52%) | 20 (58.8%) | **0.05*** |
| Subsequent 3^rd^ generation TKIs, n | 86^c^ | 1^c^ | 64 | 21^c^ | 0.12 |

^c^1 patient had De Novo T790M and 1 patient had T790M negative received osimertinib

**Table S5.** Pattern of disease progression after EGFR TKI treatment

| **Characteristics** | **All**  **(N = 332)** | **De novo resistance**  **(N = 28)** | **Intermediate responders**  **(N = 240)** | **Long-term responders**  **(N = 64)** | ***P* values** |
| --- | --- | --- | --- | --- | --- |
| Pattern of PD, n (%)  Systemic PD  Oligo-PD  CNS PD  N/A | 170 (51.2%)  72 (21.7%)  44 (13.3%)  45 | 16 (57.1%)  5 (17.9%)  3 (10.7%)  4 | 140 (58.3%)  41 (17.1%)  33 (13.8%)  26 | 14 (21.9%)  26 (40.6%)  8 (12.5%)  15 | **<0.001*** |

**Table S6.** Response Pattern and Survival

|  | **All**  **(N = 332)** | **De novo resistance**  **(N = 28)** | **Intermediate responders**  **(N = 240)** | **Long-term responders**  **(N = 64)** |
| --- | --- | --- | --- | --- |
| Best ORR, n (%)   - CR - PR - SD - PD - Not evaluable | 201 (60.5)  2 (0.6)  199 (59.9)  95 (28.6)  19 (5.7)  17 (5.1) | 1 (3.6)  0  1 (3.6)  6 (21.4)  19 (67.9)  2 (7.1) | 159 (66.2)  1 (0.4)  158 (65.8)  72 (30)  0  9 (3.8) | 41 (64.1)  1 (1.6)  40 (62.5)  17 (26.6)  0  6 (9.4) |
| PFS, median  (95% CI), months | 12.2  (11.0-13.3) | 2.1  (1.3-2.8) | 11  (9.9-12.1) | 34.9  (32.0-37.7) |
| OS, median (95%CI), months | 32.9  (27.9-37.8) | 7.2  (3.5-10.9) | 25.7  (22.1-29.3) | 80.5  (65.2-95.8) |

CI: confidence interval, CR: complete response, OS: overall survival, PD: progressive disease, PFS: progression-free survival, PR: partial response, SD: stable disease.

**Figure S1**. Progression free survival for EGFR TKI treatment in patients with *EGFR*-mutant NSCLC according to A. EGFR TKI response group (2.1 months for *de novo* EGFR TKI resistance, 11 months for intermediate responders (IRs) and 34.9 months for long-term responders (LTRs); *p*-value <0.0001). B. ECOG PS (12.8 months for PS 0-1, and 9.9 months for PS 2-4; *p*-value 0.009). C. Stage at diagnosis (18.5 months for recurrent disease, and 11.5 months for metastatic disease; *p*-value <0.0001). D. Number of metastatic sites (13.4 months for 1-2 sites, and 9.2 months for 3 or more sites; *p*-value <0.0001). E. *EGFR* mutation subtype (12.7 months for common mutations, and 5.3 months for uncommon mutations; P<0.0001). F. Common *EGFR* mutation subtype (12.5 months for del19, and 12.7 months for L858R; *p*-value 0.70).


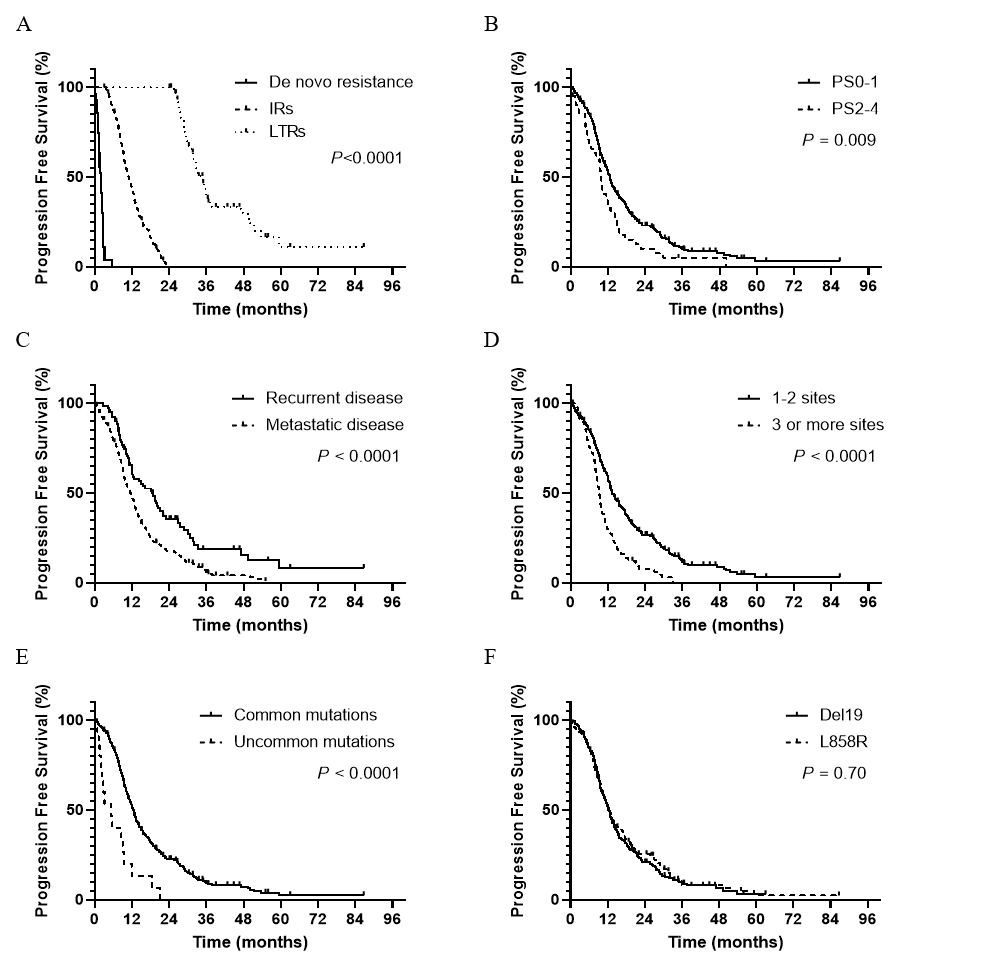


**Figure S2.** Overall survival in patients with *EGFR*-mutant NSCLC according to A. EGFR TKI response group (7.2 months for *de novo* resistance, 25.7 months for intermediate responders (IRs) and 80.5 months for long-term responders (LTRs); *p*-value <0.0001). B. ECOG PS (34.8 months for PS 0-1, and 14.9 months for PS 2-4; *p*-value <0.0001). C. Number of metastatic sites (36.9 months for 1-2 sites, and 22.8 months for 3 or more sites; *p*-value <0.0001). D. Liver metastasis (34.3 months for absence of liver metastasis, and 20.0 months for presence of liver metastasis; *p*-value 0.004). E. Treatment line of EGFR TKIs (24.6 months for first line, and 40.3 months for second or later line; *p*-value 0.006). F. Subsequent osimertinib treatment (66.2 months for subsequent osimertinib treatment, and 27.0 months for no osimertinib as subsequent treatment; *p*-value <0.0001).


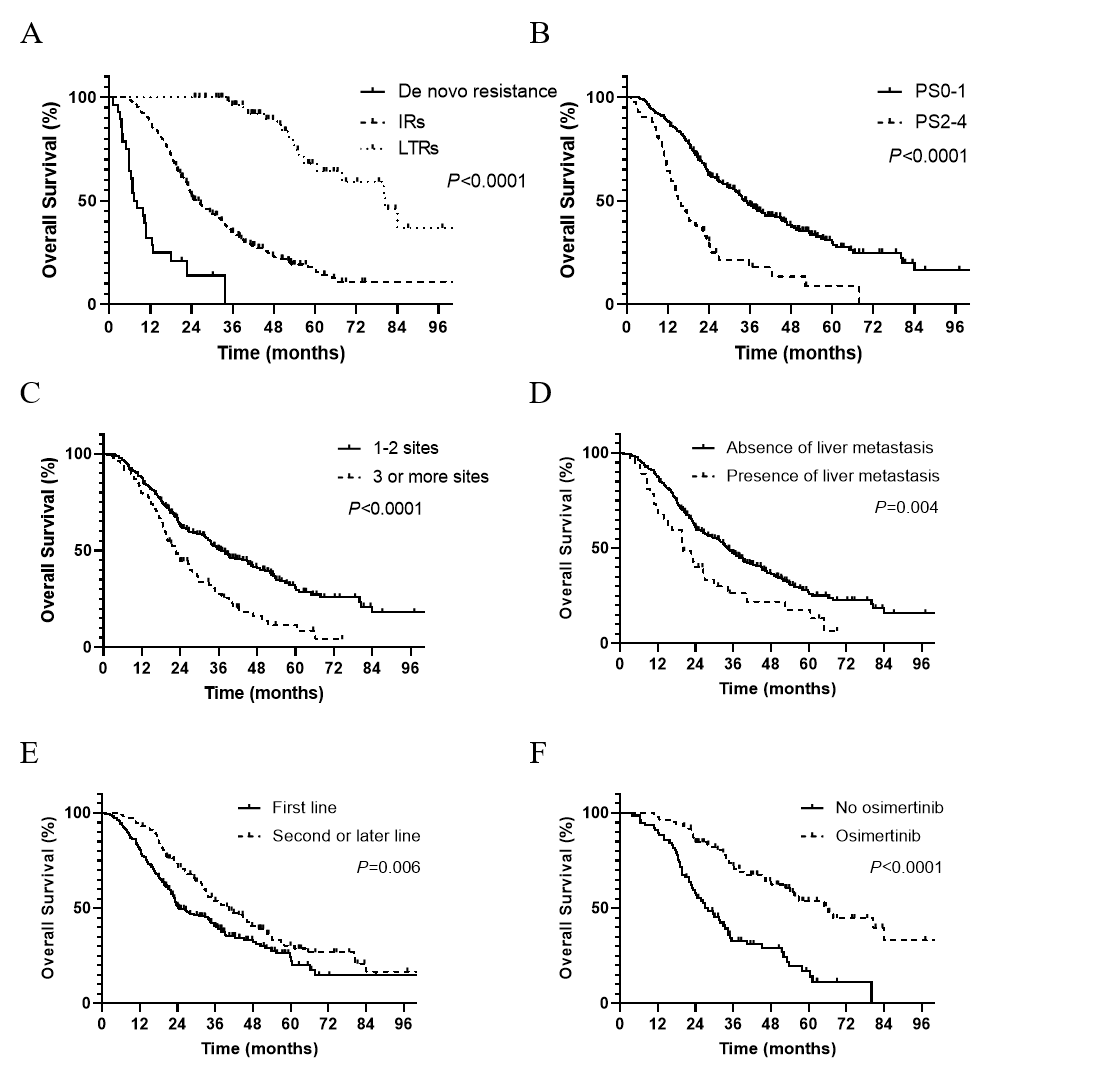


**Figure S3** waterfall plot maximal response per RECIST criteria of 48/65 advanced NSCLC by independent reviewer blind to molecular characteristic

**
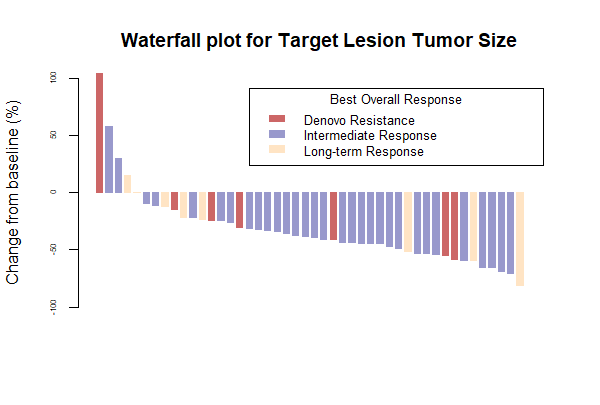
**

**Figure S4** Presence of *CDK6* amplification shown shorten PFS of EGFR TKI with the HR of PFS 2.22 [95% CI 1.24-4.0, *p-*value 0.007]. Presence of *CDK4* amplification, with less prevalence of *CDK6* amplification, also shown trend of shorten PFS of EGFR TKI with the HR of PFS 1.79 [95% CI 0.92-3.48, *p*-value 0.08].

**
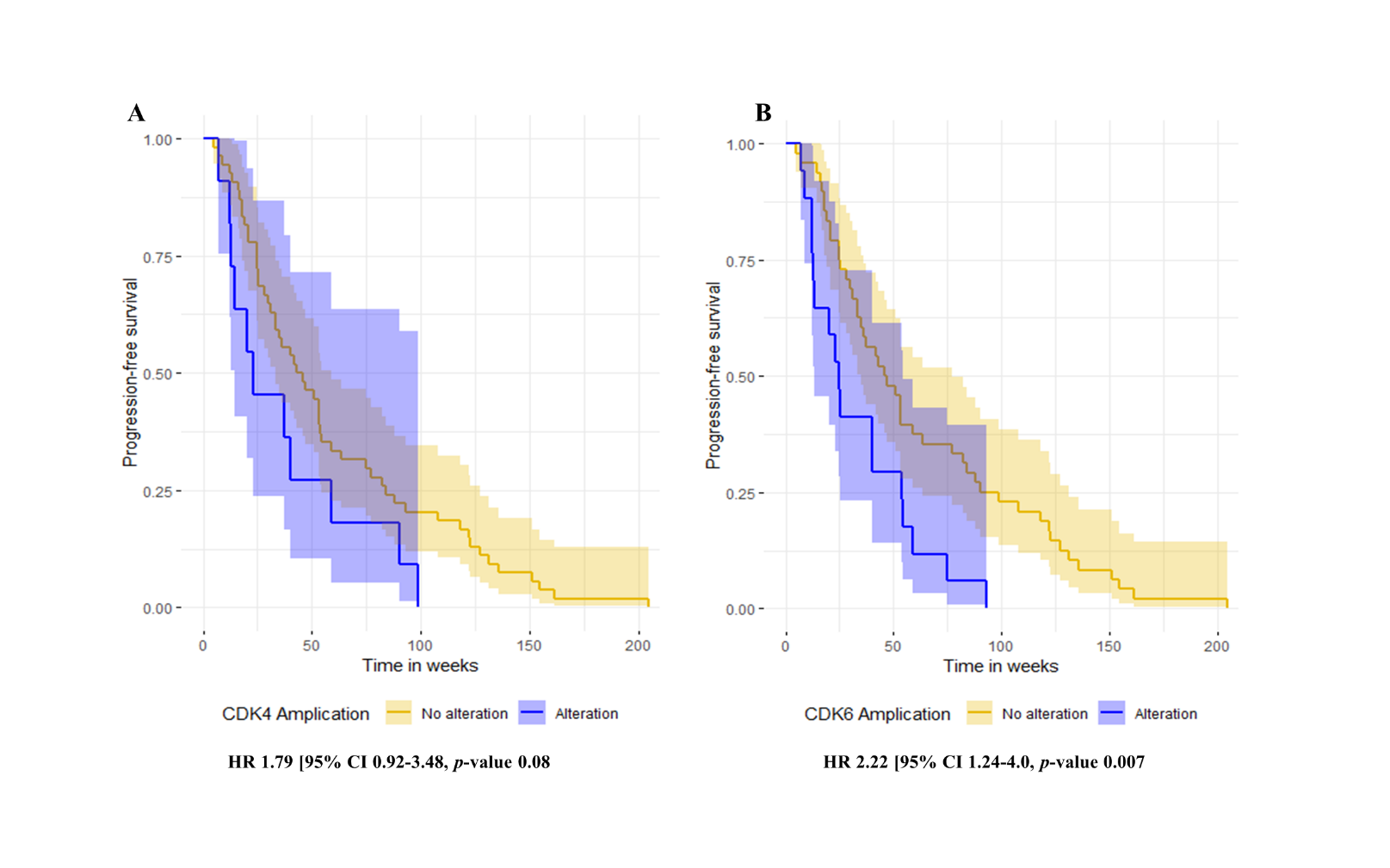
**

**Figure S5.** Mutation profile of 65 resectable non-small cell lung cancer for 21 driver genes from lung adenocarcinoma East Asian cohort using match-normal workflow WES analyses. The most frequent drivers were *EGFR* (60%), *TP53* (28%) and *RMB10* (11%) consistent with East Asian cohort (47%, 36% and 8%, respectively). While *Kras* mutation was found 5% lower than East Asian cohort (11%).


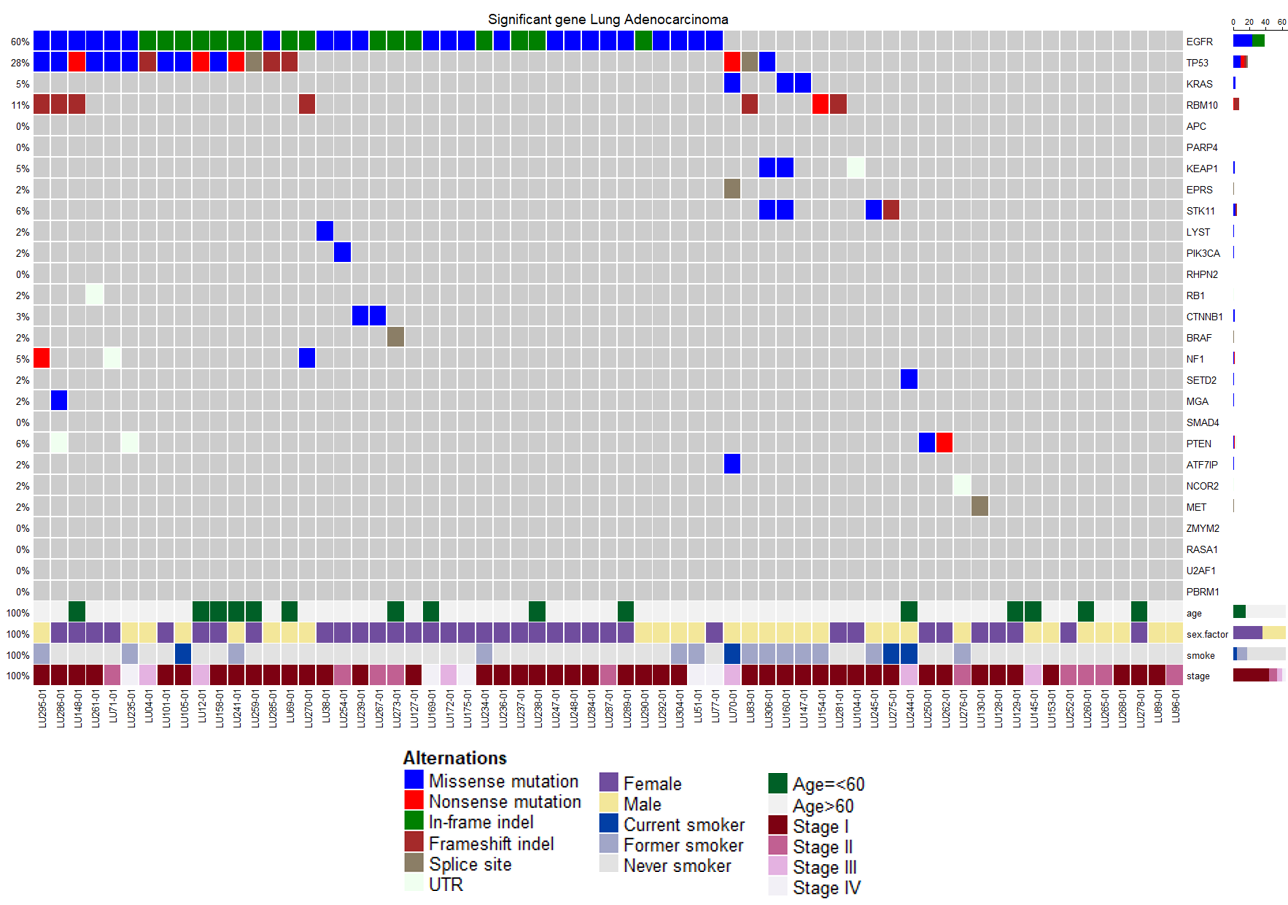


**Figure S6** Chromosome arm-level CNV frequencies of 65 resectable non-small cell lung cancer (B), pink bar represented short-arm, green bar represented long-arm. Arm with significant alterations *q*-value < 0.1 are represented in red color. Many focal CNVs were found around driver gene amplification in *EGFR*, *MYC*, *MDM2*, *KRAS* and *CCNE1* as well as deletion in *ARID1A* and *APC* consistent with East Asian cohort (A).


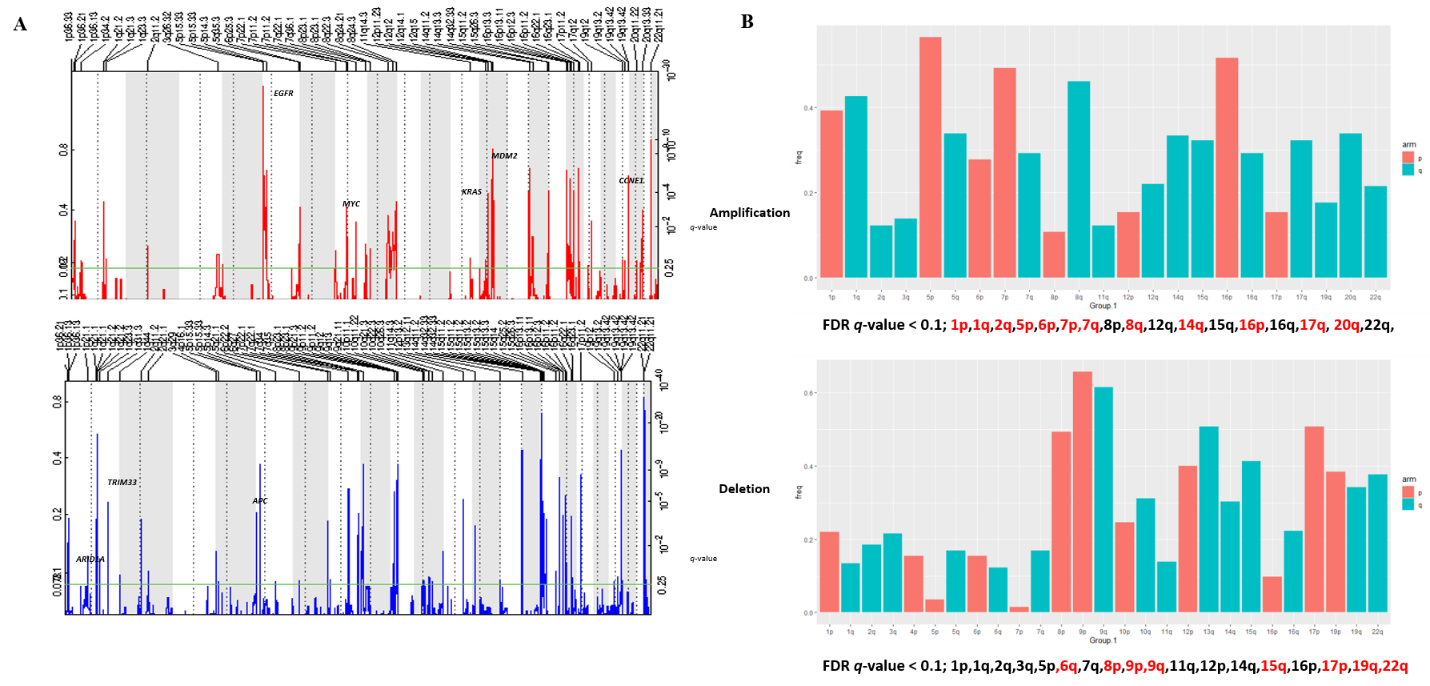


**Figure S7** Comparative of all non-synonymous variants from match-normal workflow vs. cohort-normal workflow from 65 resectable non-small cell lung cancer showed high correlation (R2=0.99, *p*-value < 2.2×10-16) of all non-synonymous mutation number between “cohort-normal” and “match-normal” workflow (B). There were 3,445 non-synonymous variants in “cohort-normal” workflow and 4,717 non-synonymous variants in “match-normal” workflow. Eighty-four percent of all non-synonymous mutations in “cohort-normal” concordance to 61.3% of “match-normal” workflow. The concordant rates in “cohort-normal” were 89% and 92.3% in 307 significant genes from LUAD 7 studies and 206 genes from 10 significant pathway analysis, respectively (A).


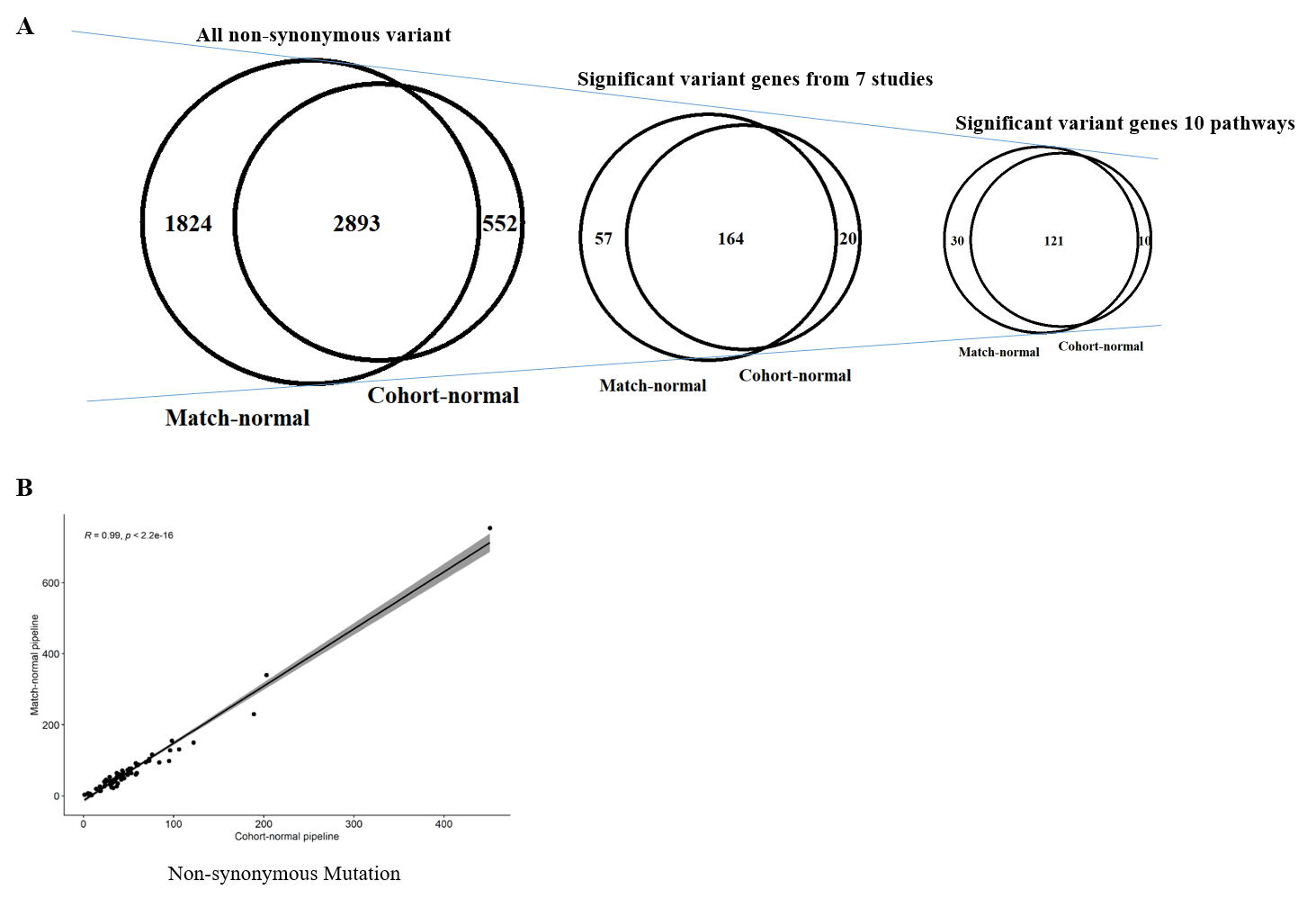


**Figure S8** Flowchart illustrating cohort-normal workflow to verify the in *silico* prediction of somatic variants. The retain variants of 65 recurrence or advanced stage EGFR-mutation positive NSCLC WES according to the filter algorithm is shown.


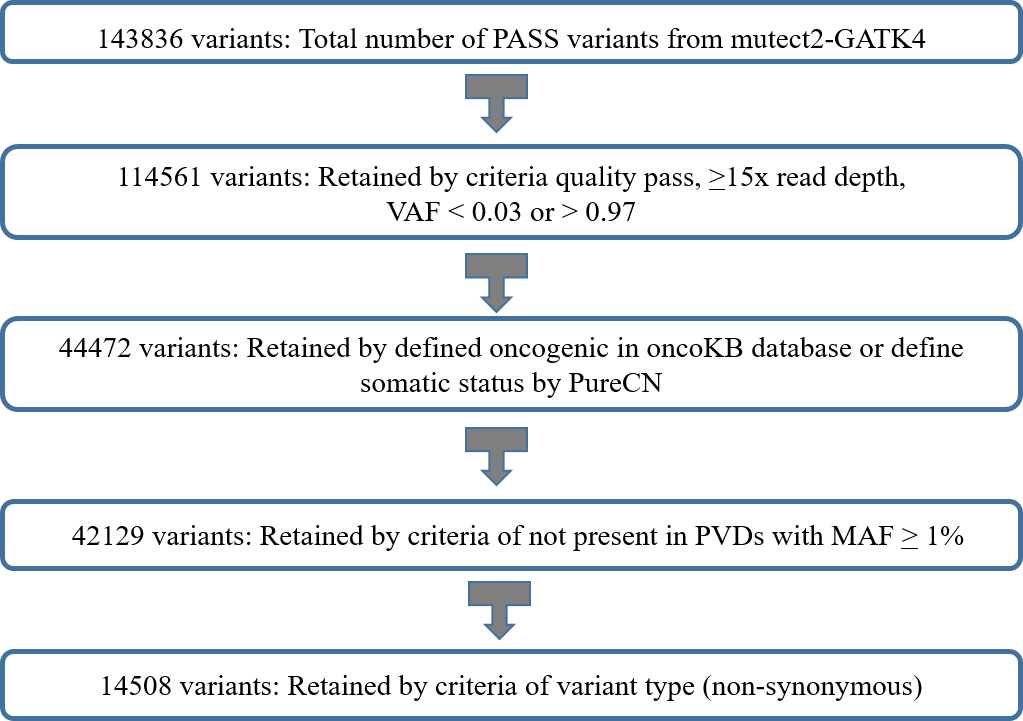


**Figure S9** Frequency of non-synonymous mutation per Mb (A), ploidy (B) and purity (C) and average depth of sequencing. The median frequency of non-synonymous mutation in de novo resistance was 1.15 Mb-1 (range: 0.65-3.33 Mb-1) lower than IRs and LTRs which was 2.82 Mb-1 (range: 1.07-6.02 Mb-1, p-value < 0.001) and 1.77 Mb-1 (range: 1.18-2.98, p-value 0.01). However, this might affect the lower average read depth in de novo resistance than IRs and LTRs, 53x (range: 40-63x), 68x (range: 60-94x, *p*-value < 0.001). No statistical difference in tumor ploidy nor tumor purity among the 3 groups.
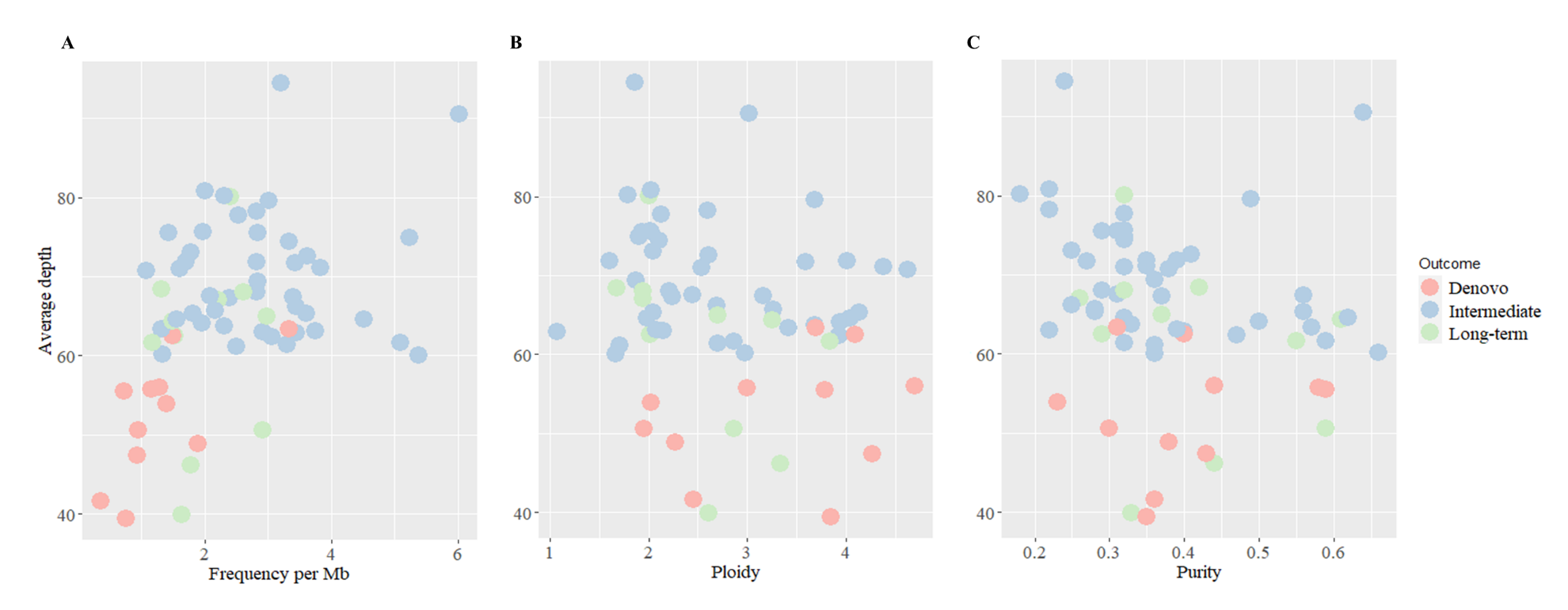


**Figure S10** Clinical factors and genomic alteration in each pathway; cell cycle (A), Hippo (B), Myc (C), Notch (D), Nrf2 (E), PI-3-Kinase/Akt (F), RTK-RAS (G), TGFβ signaling (H), p53 (I) and β-catenin/Wnt (J). The frequency of 10 pathway alterations was in range of 18-84% (18.4% NRF2, 27.6% TGF-β, 30% MYC, 52.3% WNT, 56% NOTCH, 60% cell cycle, 76.9% RTK-RAS, 69% HIPPO, 83% PI3K, and 84% TP53 pathway).

A. cell cycle pathway


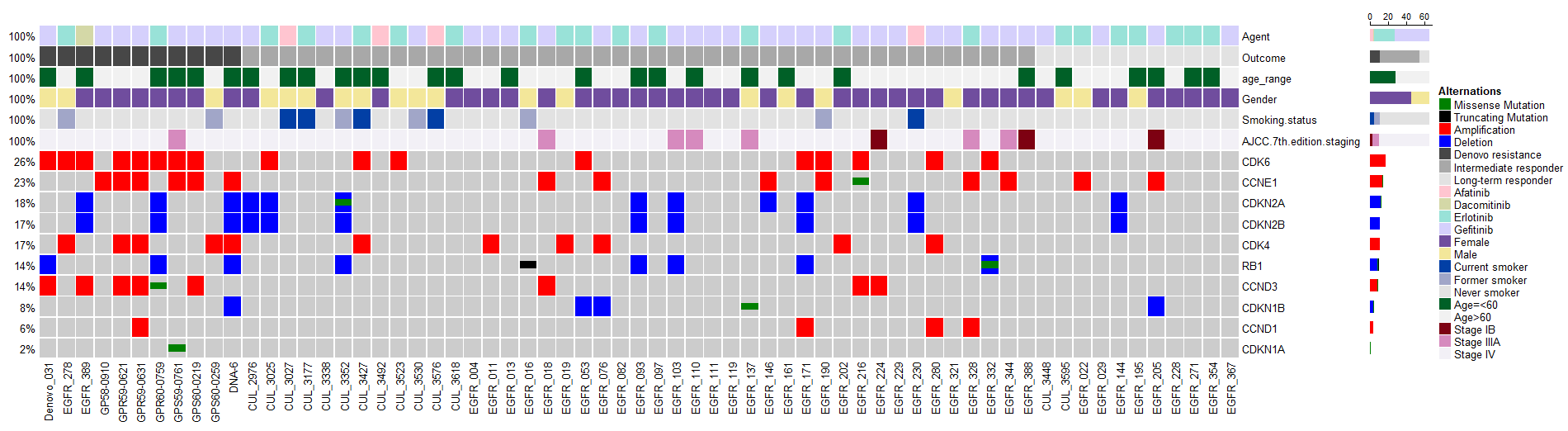


B. Hippo pathway


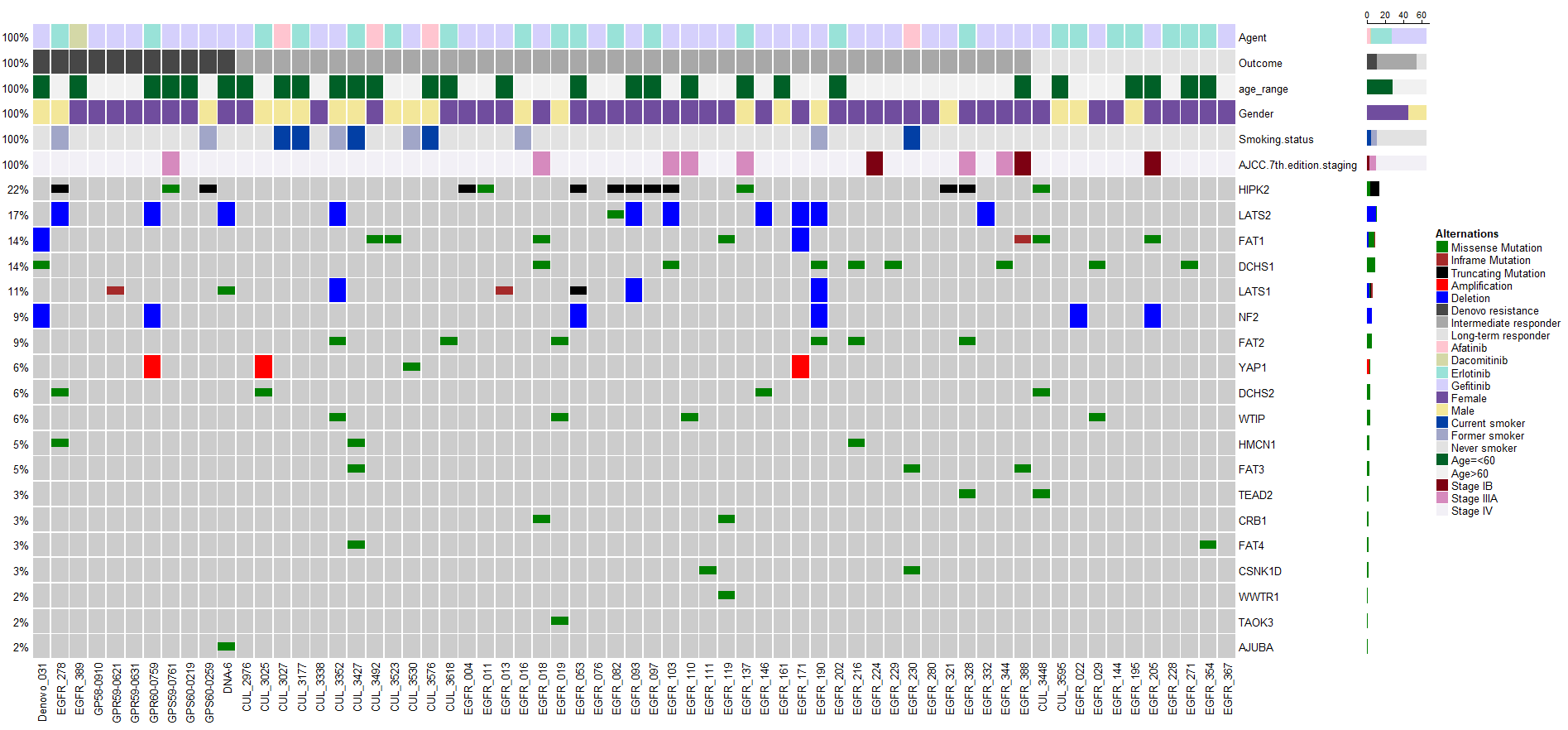


C. Myc pathway


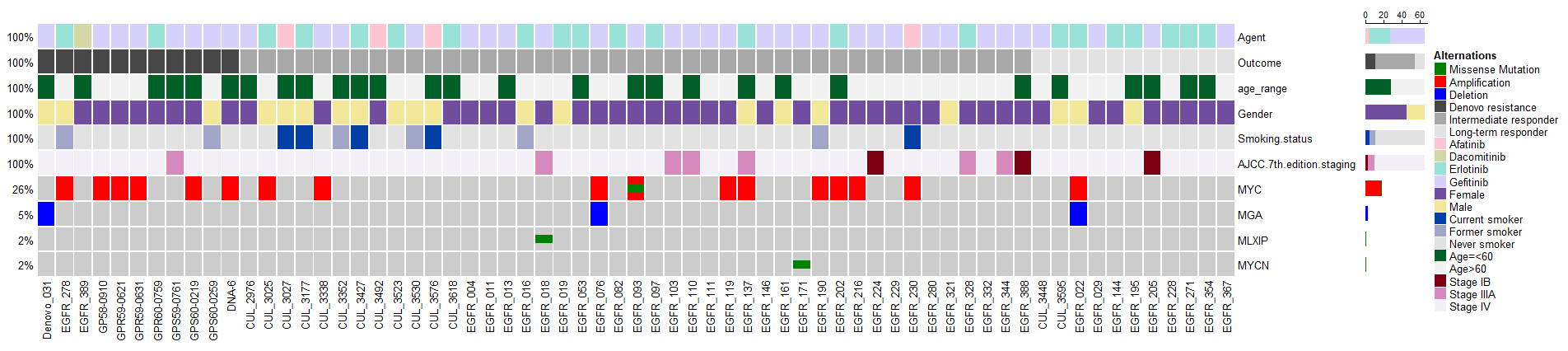


D. Notch pathway
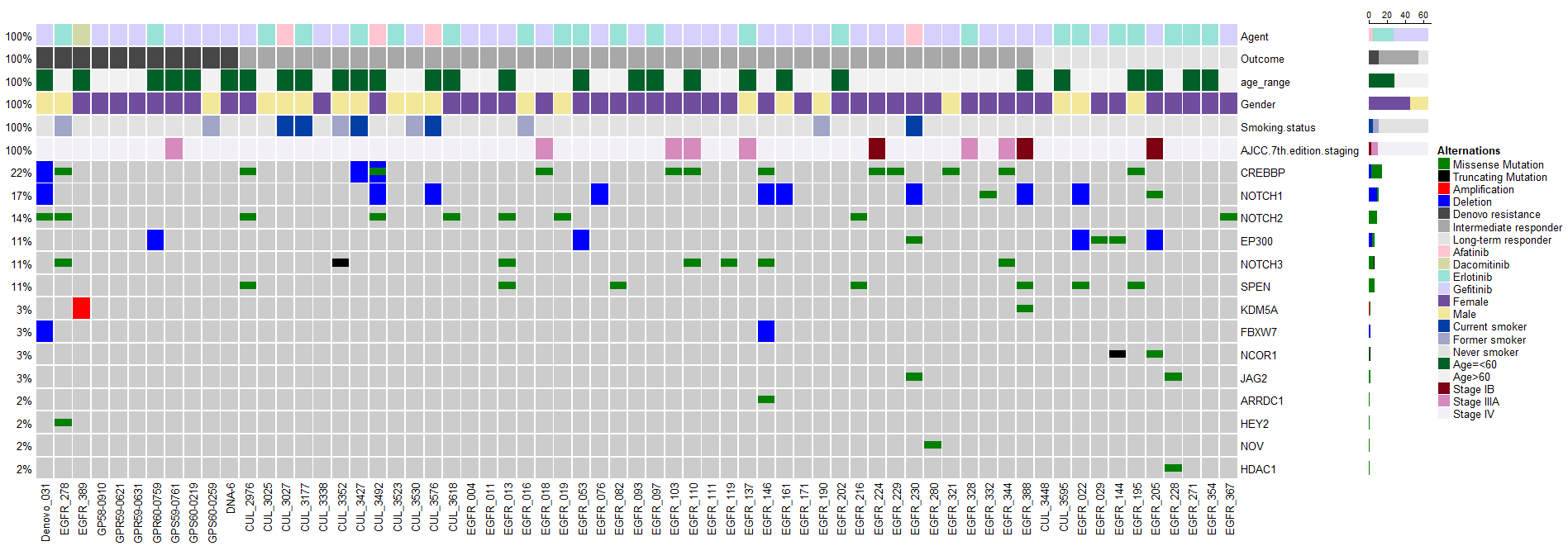


E. Nrf2 pathway


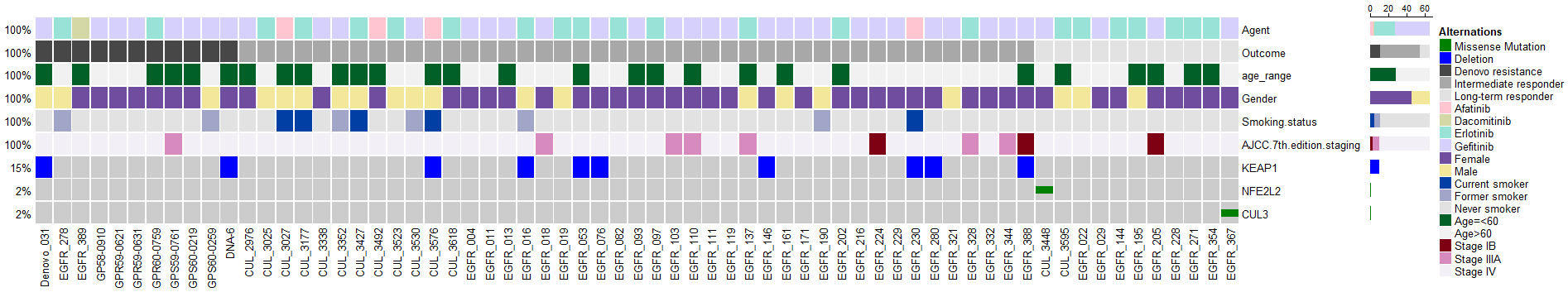


F. PI-3-Kinase/Akt pathway


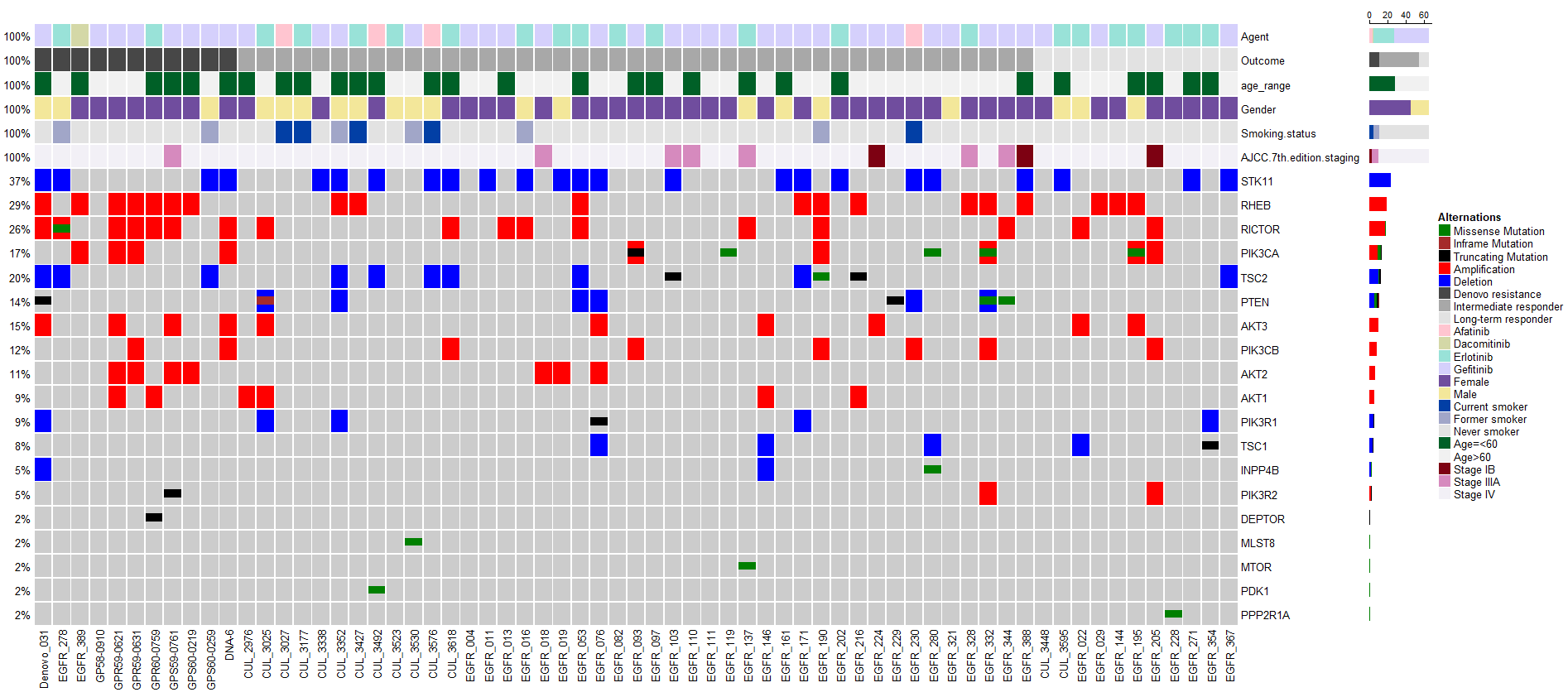


G. RTK-RAS pathway


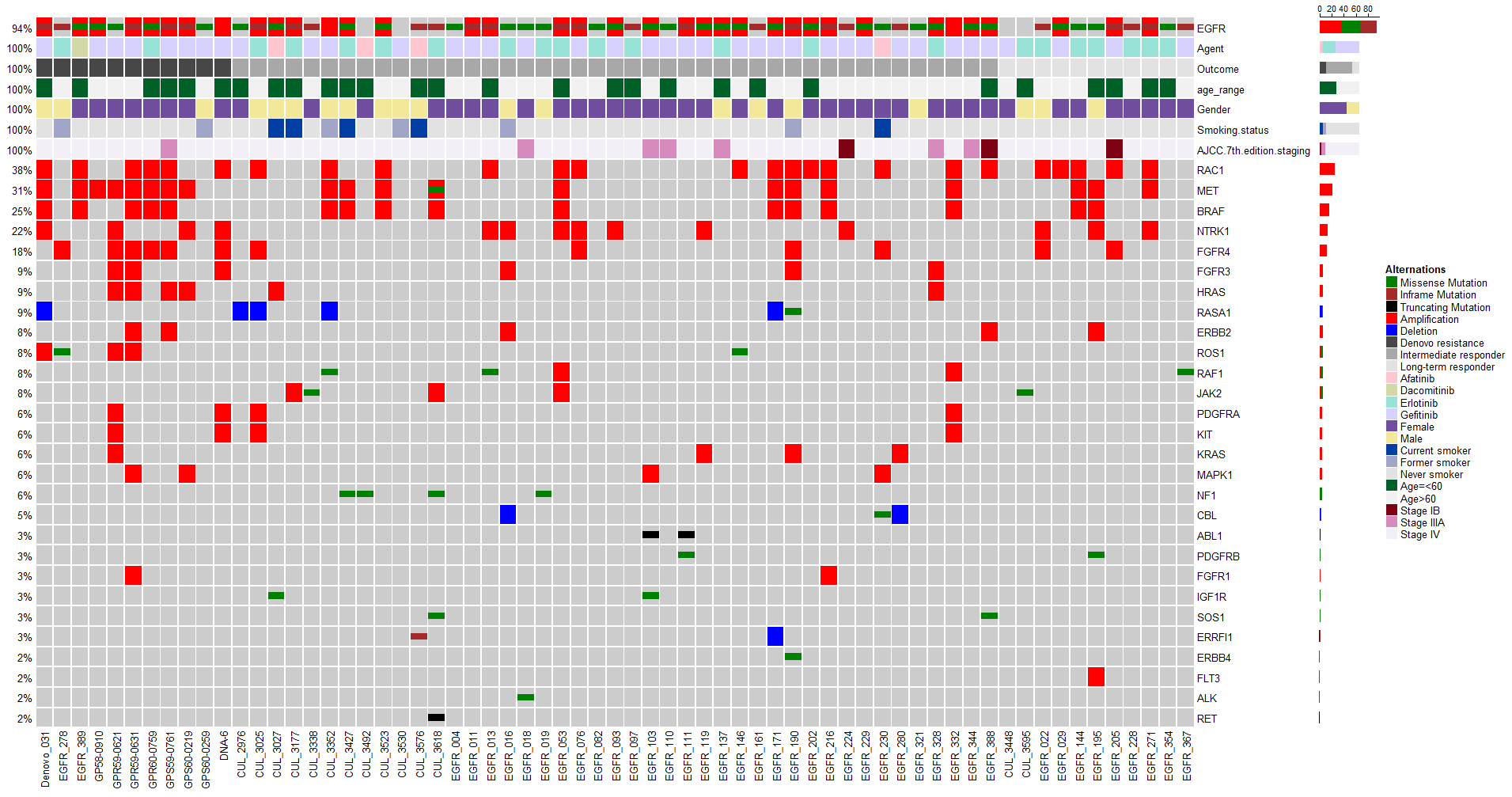


H. TGFβ signaling pathway


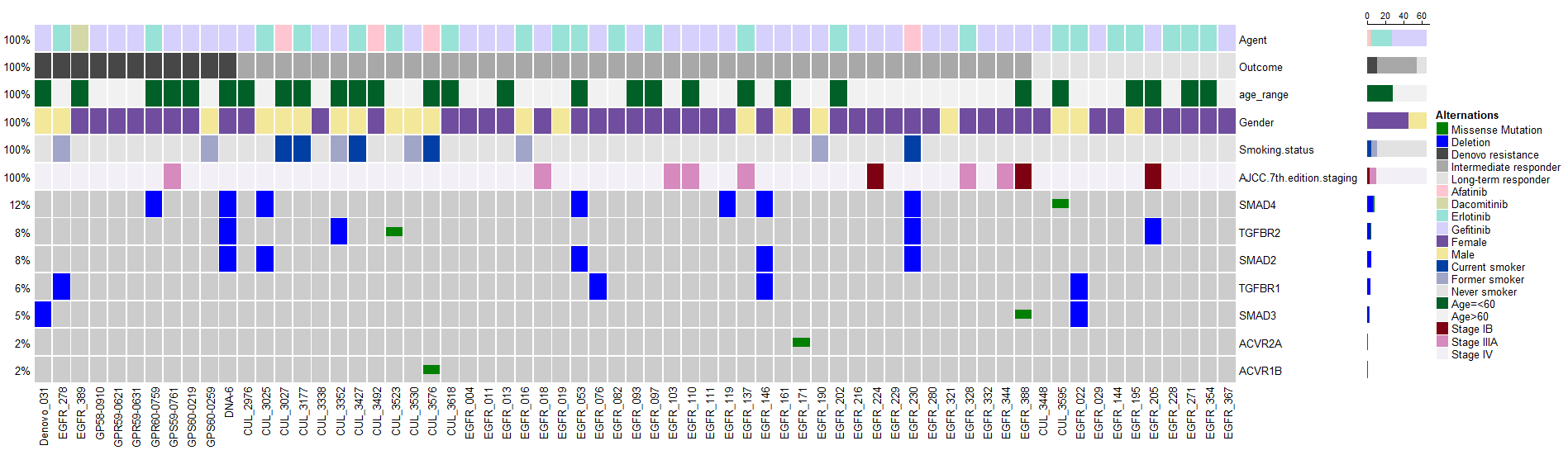


I. p53 pathway


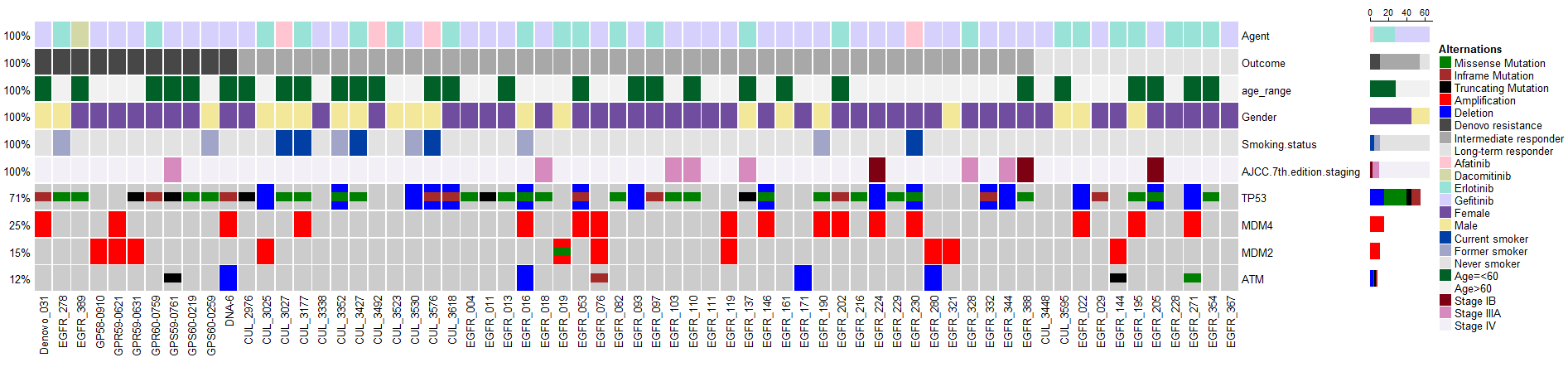


J. β-catenin/Wnt pathway


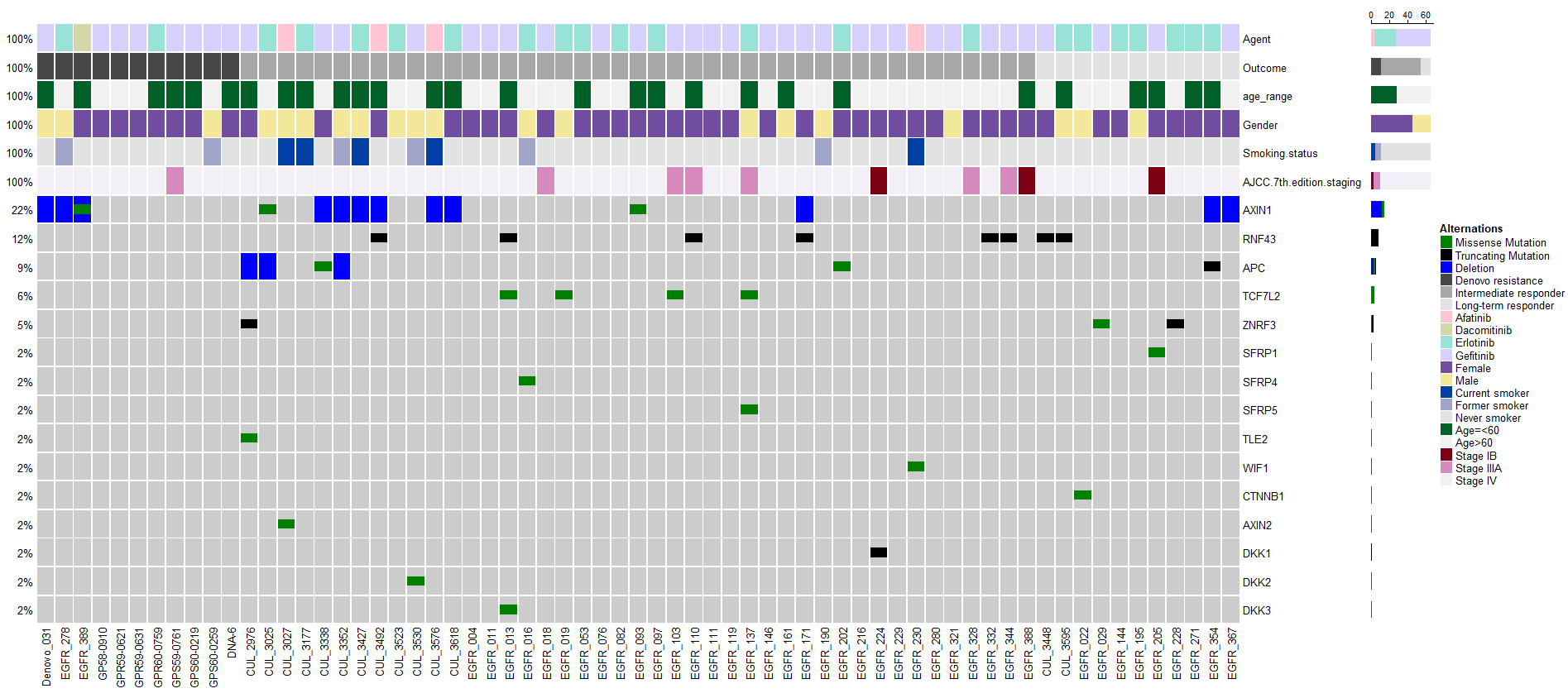

Supplement: Supplementary file 2 — Supplementary Information 2. [file 41598_2022_6239_MOESM2_ESM.docx]
